# Combined miRNA and mRNA Signature Identifies Key Molecular Players and Pathways Involved in Chikungunya Virus Infection in Human Cells

**DOI:** 10.1371/journal.pone.0079886

**Published:** 2013-11-21

**Authors:** Tanvi Saxena, Bhavna Tandon, Shivani Sharma, Shibu Chameettachal, Pratima Ray, Alok R. Ray, Ritu Kulshreshtha

**Affiliations:** 1 Department of Biochemical Engineering and Biotechnology, Indian Institute of Technology, Delhi, India; 2 Department of Textile Technology, Indian Institute of Technology, Delhi, India; 3 Department of Pediatrics, All India Institute of Medical Sciences, New Delhi, India; 4 Centre for Biomedical Engineering, Indian Institute of Technology, Delhi, India; 5 All India Institute of Medical Sciences, New Delhi, India; University of Hong Kong, Hong Kong

## Abstract

Since its discovery, Chikungunya fever caused by a virus (CHIKV) has ravaged most of Africa and Southeast Asia. Despite there being more than a million reported cases in India alone and the seriousness of the disease in the chronic phase, a clear understanding of the disease pathogenesis and host response remains elusive. Here, we use microarray technology and quantitative PCR method to establish the complete miRNA, snoRNA and mRNA signature of host response upon CHIKV infection in human cell line infection model, HEK293T. The results were further validated in human primary cells (dermal fibroblasts). miRNA expression profiling revealed regulation of 152 miRNAs post CHIKV infection. An interesting overlap in miRNA signature was seen majorly with HCV, HPV and HIV1 virus. The microarray data further validated by qRT-PCR revealed induction of miR-744, miR-638, miR-503 and others among the top upregulated miRNAs. Notably, we found induction of snoRNAs belonging to C/D cluster including close paralogs of U3, U44, U76 and U78 snoRNAs. Genes were found to be differentially expressed along 3 major pathways; TGF-β, endocytosis and the cell cycle pathways. qRT-PCR data confirmed strong induction of TGF-β (SMAD6, JUN, SKIL) and endocytosis pathway (CXCR4, HSPA8, ADRB1) genes while downregulation of cell cycle genes (CDC27 and CDC23). Interestingly, use of TGF-β inhibitor, SB-431542, increased CHIKV mediated cell death. Overall, this study aims at providing the first complete transcriptome signature of host response upon CHIKV infection to aid identification of possible biomarkers and therapeutic targets.

## Introduction

Chikungunya virus (CHIKV) is an enveloped, positive sense, single stranded RNA virus of the *Togaviridae* family and Alphavirus genus [1]. It is an insect-borne virus that is transmitted to humans by virus carrying *Aedes* mosquito though a few cases of maternal-fetus transmissions have been reported [2], [3]. The incubation period for CHIKV ranges from 3 to 7 days, in accordance with other alphaviruses. The classic clinical symptoms post CHIKV infection are abrupt febrile illness, polyarthralgia and maculopapular rash [4], [5]. However, ∼5% of CHIKV cases remain asymptomatic [4]–[8]. Due to similar initial symptoms, Chikungunya is often confused with Dengue, but the characteristic arthralgia in its chronic phase differentiates the two [9]. Until now, there is no effective treatment for the disease and the patient management is mainly symptomatic via analgesics and primary anti-inflammatory drugs [10]. This is primarily because of critical lack of knowledge about mechanism of CHIKV pathogenesis and the molecules involved.

Viral infections involve active dynamics between the host cell and the virus particle. Virus utilizes host machinery in various ways to its advantage while host cell activates the innate and adaptive responses to block viral replication and thus the spread [11]. Therefore, designing specific viral therapeutics and diagnostics requires identification of key viral and host factors that are required for viral entry and infection. Until now there is no comprehensive study on molecular interactions involved in CHIKV: host biology. Recently, cytokines and chemokines were shown to play an important role in CHIKV immunopathology [12]. Although Interferon -γ, Interleukin-2 and Interleukin-10 have been shown to be involved in the pathogenesis of CHIKV, their role is still unexplored and limited studies have been conducted on the virus' capability to induce cell damage and secretion of associated factors [13]. Certain studies implicating Interleukin–1β, Interleukin–6, and RANTES (**R**egulated on **A**ctivation, **N**ormal **T** cell **E**xpressed and **S**ecreted) as biomarkers of Chikungunya severity were conducted but their roles were not investigated in detail [14]. Many studies concerning the chronic stage of CHIKV infection and the autoimmune nature of the subsequent arthralgia have been conducted [4], [5] but the study of the initial host response is surprisingly bleak. The mobilization of the apoptotic machinery by CHIKV for propagation has been reported which is indicated in elevated caspase levels post infection [15]. Altogether, even though studies elaborating upon the structure of the virus itself have been numerous, a complete picture of the host response to CHIKV infection has not yet been reported.

Cellular response to infection is a dynamic process that involves complex biological responses of the host. To explain these processes at a global level, we recorded changes in gene expression of both coding genes and microRNAs (miRNAs) (a category of small non-coding RNA) post CHIKV infection at various time points. miRNAs are known to be post transcriptional regulators of gene expression. They act by binding to complementary sequences in the 3′UTR of various transcripts and targeting them for degradation or translational block depending on the degree of complementarity [16]. Present estimates suggest that miRNAs may regulate upto 60% of the human genes. Consequently, deregulated miRNA expression leads to huge alterations in protein levels and thus is linked to various human pathologies like cancer, diabetes, neurodisorders and infections [17]. Even though miRNAs have been found to be involved in many viral infections, no studies investigating the effect of CHIKV infection on the miRNA levels has yet been reported. In our study, using miRNA and gene expression profiling studies and qRT-PCR data, we establish the complete mRNA and miRNA signature of host response to CHIKV infection. This will be significant in inferring CHIKV pathogenesis and identification of possible biomarkers and therapeutic targets.

## Materials and Methods

### Cell Lines and Virus

HEK293T, Vero-76 and primary human dermal fibroblast cells (passage 2) were maintained in DMEM (Cell Clone) media supplemented with 100 U/ml penicillin, 100 ug/ml streptomycin and 10% fetal bovine serum and incubated at 37°C in 5% CO_2_ in an incubator. The primary human dermal fibroblasts were a kind gift from Dr. Sourabh Ghosh (Indian Institute of Technology, Delhi). The CHIKV virus (African strain) used was obtained from National Institute of Virology, Pune.

### Virus Detection by RT-PCR

Forward & reverse primers were designed for the 294 base pairs partial nucleotide sequence of E1 gene. The CHIKV was detected by doing semi-quantitative RT-PCR using primers specific for E1 gene as previously described [8].

### Virus Infection

HEK293T or primary human dermal fibroblast cells were infected with the CHIKV virus at a MOI (multiplicity of infection) of 1. Cell supernatant and total RNA were collected at 0, 12 and 24 hr post infection. CHIKV virus titration was done in Vero and HEK293T cells using MTT Assay and calculated in units of TCID50/ml.

### miRNA microarray profiling and mRNA expression profiling

Total RNA isolation was conducted using TriZol reagent (Invitrogen) in accordance with manufacturer's instructions. Total RNA isolated from HEK293T cells infected with CHIKV (MOI of 1) for 0, 12 and 24 hr was sent to iLife Discoveries for miRNA and gene expression profiling. The miRNA microarray was conducted using Affymetrix Gene Chip miRNA 2.0 platform while the mRNA expression profile was generated using Affymetrix Gene Chip PrimeView platform. A cut off of fold change more than or equal to 1.5 was employed to identify the differentially regulated miRNAs and mRNAs (**Data S1 and S2**). The microarray data has been submitted to GEO repository. The accession numbers are GSE49985 (gene expression data) and GSE49984 (microRNA expression data).

### Stem Loop RT-PCR and qRT-PCR

Stem-loop RT-PCR was done to determine the level of specific miRNAs in all the samples for validation of microarray data. RNU6B was used for normalization of the data. First, cDNA was made with RT primer specific for the miRNAs or U6 using RivertAid First strand cDNA synthesis kit (Fermentas). Subsequently, real-time PCR was performed using specific forward and a common Stem-loop reverse universal primer and Power SYBR Green master mix kit (Ambion).

Similarly, for the validation of gene expression profile data, isolated RNA was reverse transcribed using Rivertaid First strand cDNA synthesis kit (Fermentas) and the cDNA formed was further amplified using primers specific to selected genes and Power SYBR Green master mix kit (Ambion). GAPDH (Glyceraldehyde 3-phosphate dehydrogenase) was used for the normalization of the data. The primer sequences used for amplification of specific miRNAs or mRNAs are given in **Data S3**. The qRT-PCR experiments were performed as three biological replicates with three technical replicates each.

### Target Prediction, Inverse correlation and Pathway Analysis

The probable targets for the differentially regulated miRNAs were selected on the basis of prediction by established target prediction programs. The targets were selected if they were found to be predicted by at least 5 target prediction programs out of 11 available target prediction programs TargetScan, PITA, PicTar, miRanda, mirTarget2, NBmirTar, RNAhybrid, MiTarget, MicroInspector, RNA22, and DIANA, MicroT, using the online software, miRECORDS [18] (**Data S7**). The list was matched with the gene expression profile data and genes displaying inverse correlations were listed separately (**Data S8**).

Pathway and Functional analysis for the differentially regulated genes and the genes showing inverse correlations was done using Database for Annotation, Visualization and Integrated Discovery (DAVID) Gene Ontology tool (http://david.abcc.ncifcrf.gov/) available online with PANTHER and KEGG Pathways as the selected databases [19].

### TGF-β Receptor Inhibitor

Solid anhydrous SB-431542 was dissolved at a concentration of 26 mM in DMSO. SB-431542 was added to cells at a final concentration of 5 µm or 10 µm. Cells were infected with CHIKV at an MOI of 0.5 and MTT Assay was performed to compare the progression of CHIKV infected cells treated with SB-43152 versus non-treated ones.

## Results

### miRNA expression signature

**Figure 1:**
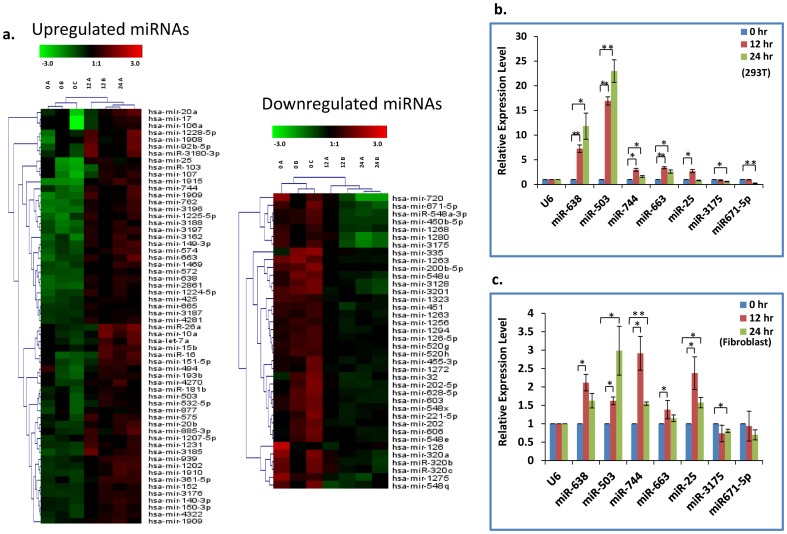
The microRNA signature of CHIKV. (**A**) Hierarchial clustering of differentially expressed miRNAs (>1.5 fold, p<0.05) commonly regulated at 12 and 24 hr as compared to 0 hr control in response to CHIKV infection in HEK293T cell line. A and B refer to the biological replicates. (**B**) Real-time PCR data showing validation of the microarray results for differentially regulated miRNAs in HEK293T cells. (**C**) Real-time PCR data showing validation of the microarray results for differentially regulated miRNAs in primary human dermal fibroblasts. The graphical data points in B and C represent mean+S.D of at least three independent experiments. (*P<0.05, **P<0.01). Error bars denote+SD.

**Figure 2:**
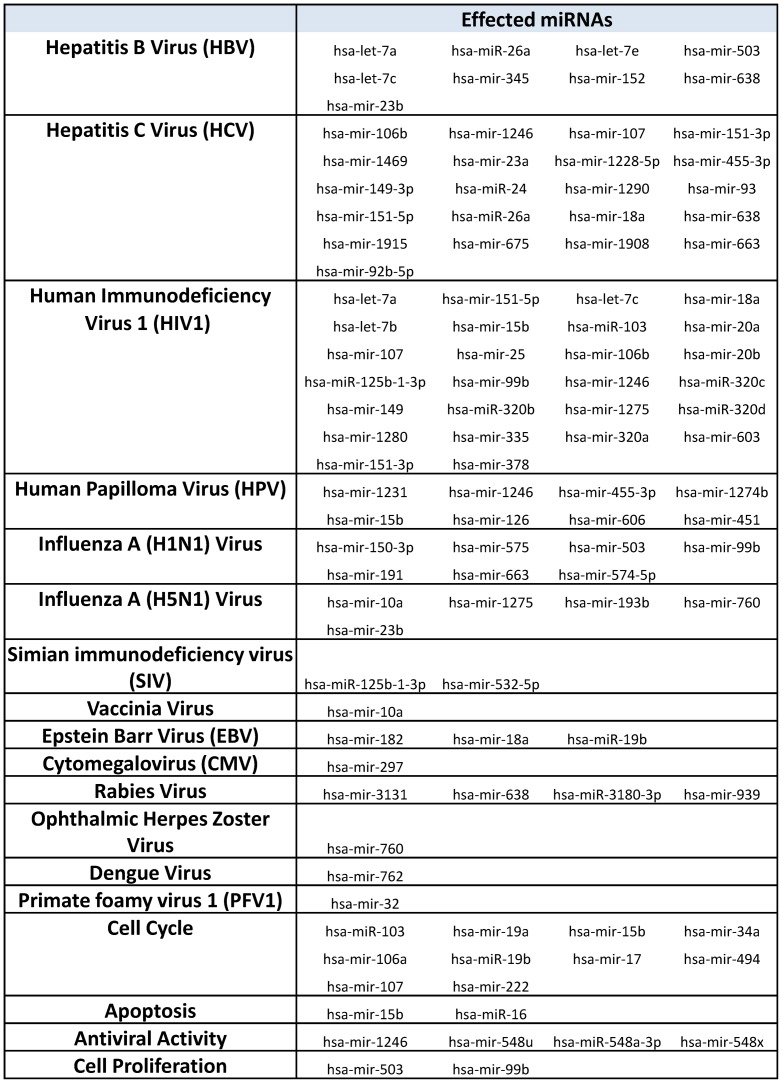
List of CHIKV regulated miRNAs implicated in other virus pathogenesis. Literature survey was conducted to find the miRNAs which have previously been reported to be involved in viral pathogenesis or associated pathways.

To identify the miRNAs involved in CHIKV infection and pathogenesis, total miRNA expression was profiled in human embryonic kidney cell line, HEK293T, following CHIKV infection. HEK293T, an epithelial cell line, has been previously reported to be highly susceptible to CHIKV [20]. HEK293T cells were infected with CHIKV at a multiplicity of infection (MOI) of 1 and total RNA samples were isolated from the infected cells at 0, 12 and 24 hr post infection. miRNA expression profile was determined using microarray technology (Affymetrix platform). The number of differentially regulated miRNAs was determined by a fold change threshold of 1.5 (P-Value<0.5) over the control sample (0 hr CHIKV infection) (**Data S1**). Out of a total of 152 differentially regulated miRNAs, 65 were up and 33 were down, 12 hr post infection. Similarly, 99 were up and 46 were downregulated at 24 hr post infection. A total of 59 miRNAs were found to be upregulated while 33 were downregulated commonly at 12 and 24 hr post CHIKV infection (**Figure 1a** **, Data S1**). Overall most of the miRNAs show increased fold change difference at 24 hr as compared to the 12 hr sample indicating their progressive increase/decrease with increasing time post infection. Interestingly, upregulated miRNAs constitute 65–70% of the total number of differentially regulated miRNAs at both 12 hr and 24 hr post infection (**Data S1**). Experimental validation was done using quantitative RT-PCR for seven randomly chosen miRNAs (five upregulated and two downregulated) and we noticed significant induction in the levels of miR-503, -638, -663 and -744 while slight downregulation in miR-3175 and miR-671-5p (**Figure 1b**). The experiments were also conducted on primary human dermal fibroblasts and similar results were obtained with miR-503 and miR-638 showing maximal induction over the course of the CHIKV infection (**Figure 1c**). The miRNA levels were normalized against RNU6B (U6) RNA. miRNA expression profiling also revealed induction of pre-miR-663 at both the time points post infection apart from the mature miR-663 form. Similarly miR-1263 showed downregulation both at the mature and precursor level at 12 and 24 hr post infection. We next did literature survey to see if the differentially regulated miRNAs have been shown to be involved in other viral pathogenesis or associated pathways. Interestingly, about 53% of the upregulated miRNAs and 45% of the downregulated miRNAs have been implicated in other viral infections, particularly HCV, HBV, HPV and HIV1 (**Figure 2** **, Data S4**). A CHIKV specific miRNA signature that includes only those miRNAs that are specific for CHIKV infection in human cells is shown in **Data S5**.

Several human miRNAs are expressed as a cluster. We thus wanted to investigate the pattern of expression of clustered miRNAs particularly with respect to recent findings showing differential expression pattern of members of many of these clusters.Interestingly, several miRNA clusters were observed among the upregulated miRNAs. These include members from the miR-17-92, let-7e/99b, miR-191/425, miR-106b/miR25, miR-23a/24 and miR-15b/16 clusters (**Data S6**). This suggests that these miRNA clusters are co-regulated in response to CHIKV infection. Also, among the induced miRNAs expressed as clusters, we confirmed upto 33 fold induction in hsa-miR-17 using quantitative RT-PCR. Other members of the clusters were also tested and induction confirmed (**Data S6**).

### snoRNA expression signature

**Figure 3:**
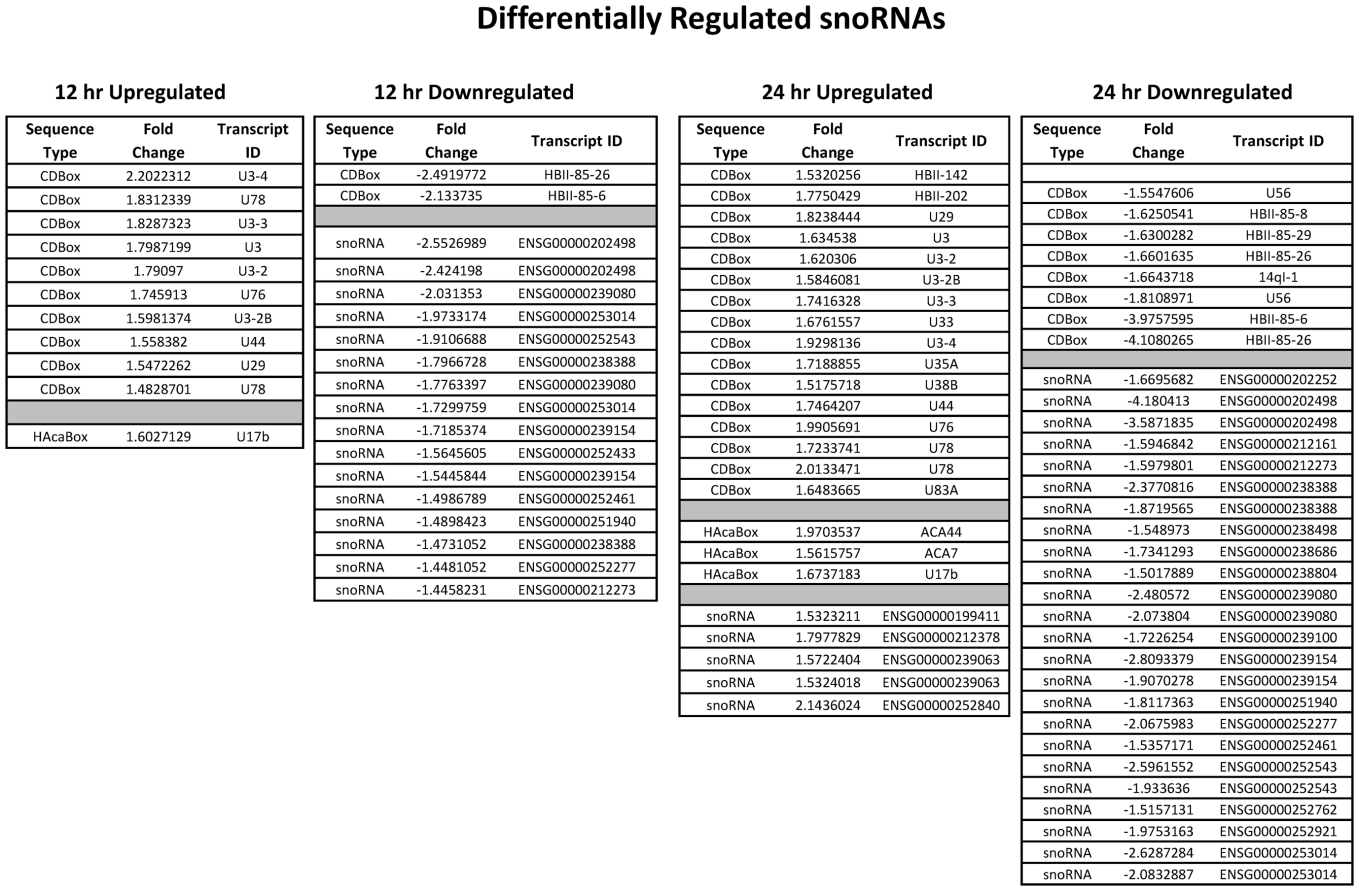
The snoRNA signature of CHIKV. HEK293T cells were infected with CHIKV and snoRNA expression profiling was done at 0, 12 and 24

Apart from known miRNAs, a number of small nucleolar RNA (snoRNA) were also found to be differentially regulated. These metabolically stable RNAs are localized in the nucleolus and are known to be involved in rRNA processing and ribosome assembly. These can be categorized into 3 main groups - C/D box, H/ACA box and the RNA component of RNase MRP (Mitochondrial RNA Processing endoribonuclease) [21], [22]. Out of the 48 upregulated snoRNA (12 hr and 24 hr post infection), 16 were C/D box type (**Figure 3**). These are known to be involved in rRNA complementarity and site specific ribosome methylation. The number of differentially regulated H/ACA box snoRNA is either very little or none at all. The rest of the snoRNA can either belong to the third group or be involved in tRNA processing or can be yet unknown (orphan snoRNAs).

### Gene expression signature

To study the changes in gene expression following CHIKV infection and to determine putative targets of the differentially regulated miRNAs, we determined the gene expression profile using microarray technology (Affymetrix platform) at 12 hr and 24 hr post infection. Genes showing more than 1.5 fold change (p<0.5) were considered to be differentially expressed. A total of 1217 genes were found to be induced while 988 genes were downregulated at 12 hr while 1187 genes were induced and 1018 were downregulated at 24 hrs post CHIKV infection. Of these, 577 genes were commonly induced at both the time points while 951 were downregulated (**Data S2**).

**Figure 4:**
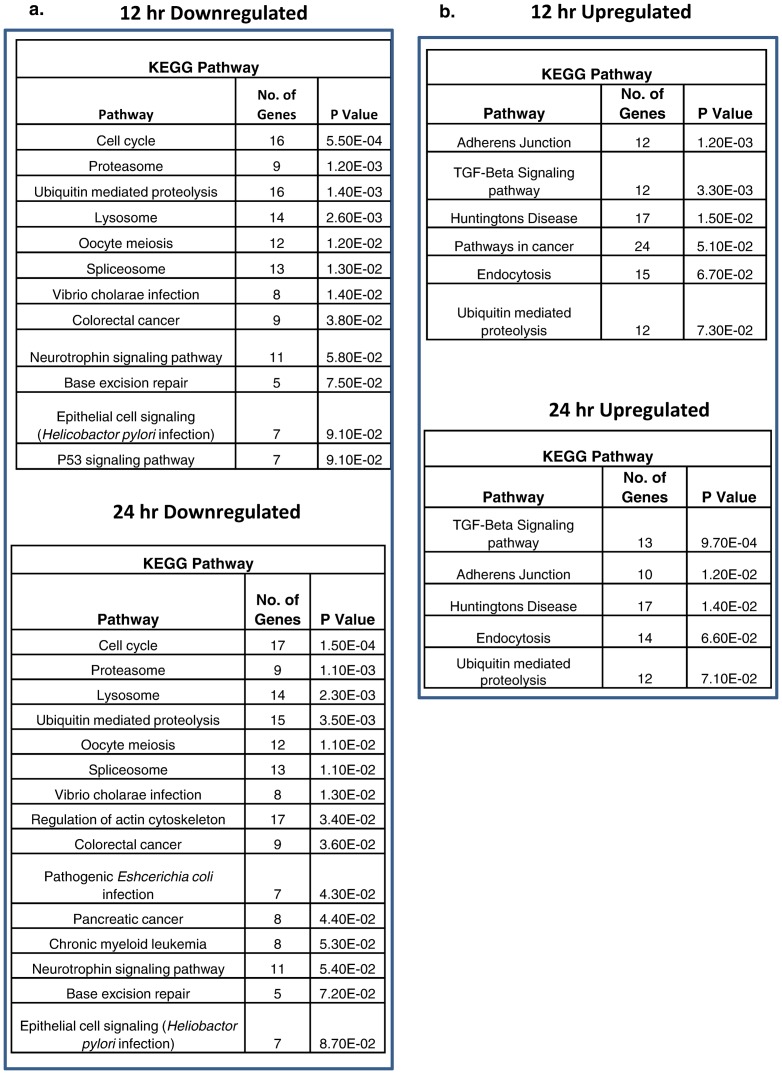
Pathway analysis of CHIKV regulated genes. The differentially regulated genes (>1.5 fold) were placed into different pathways based on analysis by DAVID software. The tables show the distribution of genes (**A**) downregulated by CHIKV and (**B**) upregulated by CHIKV.

**Figure 5:**
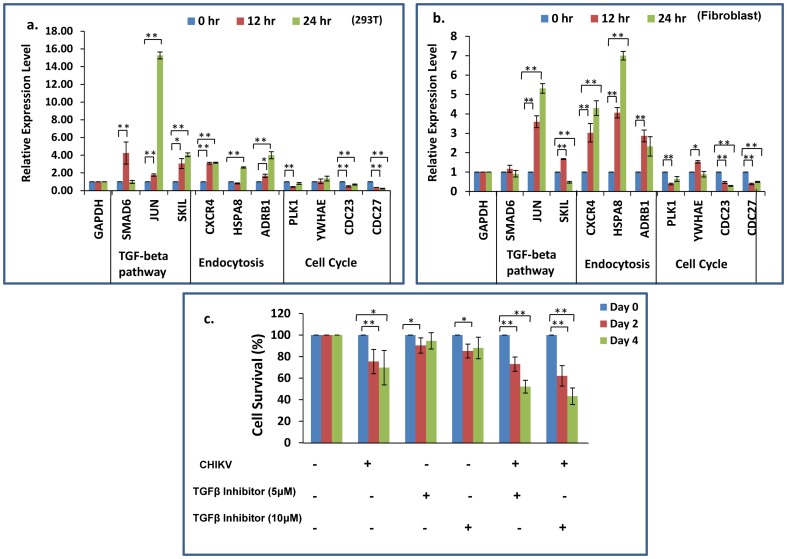
CHIKV regulates TGF-β, Endocytosis and Cell Cycle pathway genes. (**A**) qRT-PCR based validation of differential expression of genes belonging to TGF-β, endocytosis and cell cycle pathways in response to CHIKV infection in HEK293T cells and (**B**) human dermal fibroblasts (**C**) Effect of TGF-β receptor inhibitor on the progression of CHIKV infection in HEK293T cells. The graphical data points represent mean+S.D of at least three independent experiments. (*P<0.05, **P<0.01). Error bars denote + SD.

Pathway analysis was conducted using the online software DAVID, for the genes with a fold change of 1.5 or higher against the control samples. Network analysis showed that the Transforming growth factor-beta (TGF-β), endocytosis and the adherens junction pathway were significantly upregulated while the cell cycle, proteasome and lysosome pathways were downregulated (all with P< = 0.1) (**Figure 4a,b**). Genes which were highly upregulated in the TGF-β and the endocytosis pathways were further validated by quantitative RT-PCR in both HEK293T and human primary dermal fibroblasts (**Figure 5a,b**). Analysis of the downregulated genes suggest that the cell cycle pathway is significantly suppressed with 16 (out of 988) and 17 (out of 1018) genes downregulated at 12 and 24 hr post infection, respectively (**Figure 4a,b**). We validated by qRT-PCR the downregulation of the cell division cycle pathway genes (CDC27 and CDC23) in HEK293T and human dermal fibroblasts upon infection with CHIKV (**Figure 5a,b**). Polo-Like Kinase-1 (PLK1) showed downregulation at 12 hr post infection in both the cell types. Other pathways downregulated upon CHIKV infection include ubiquitin mediated proteolysis and the proteasome and lysosome associated genes.

Since our studies showed a strong induction in the genes involved in the TGF-β pathway, we wanted to analyse the effect of its inhibition on the progression of infection (**Figure 5c**). We used SB-431542, which inhibits the ACVR receptors, at a final concentration of 5 µm and 10 µm, to study its effect on dynamics of CHIKV infection in HEK293T cells. Interestingly, we found that CHIKV mediated cell death increases with increasing concentration of the inhibitor.

**Figure 6:**
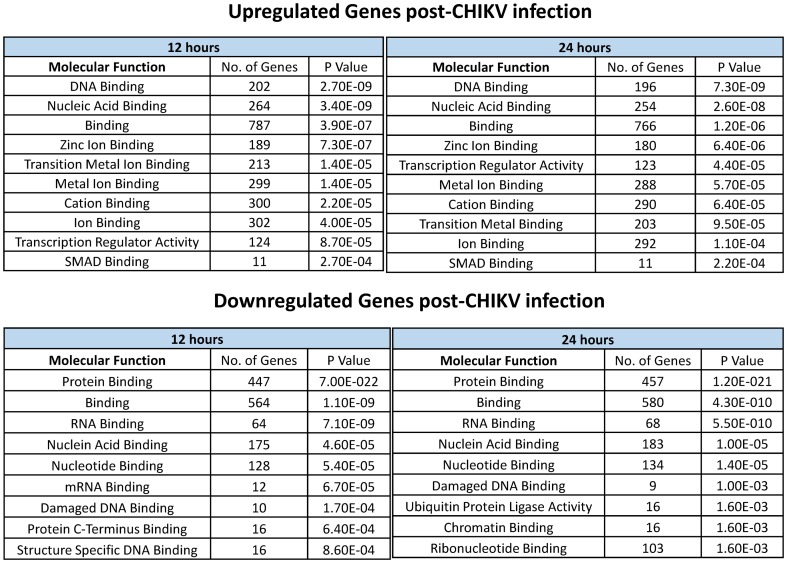
Functional enrichment analysis of CHIKV regulated genes. The differentially regulated genes (>1.5 fold) were placed into different functional categories based on analysis by DAVID software.

Functional clustering of the induced genes, done using DAVID, show that at 12 and 24 hr post infection, most of the genes are involved notably in DNA binding, transcription factors, Zinc finger transcription factor and Sma- and Mad-Related Proteins (SMAD) binding (**Figure 6**). Major functional clusters among the downregulated genes, at both 12 and 24 hr, were the genes involved in protein and RNA binding (**Figure 6**).

### miRNA∶mRNA crosstalk

**Figure 7:**
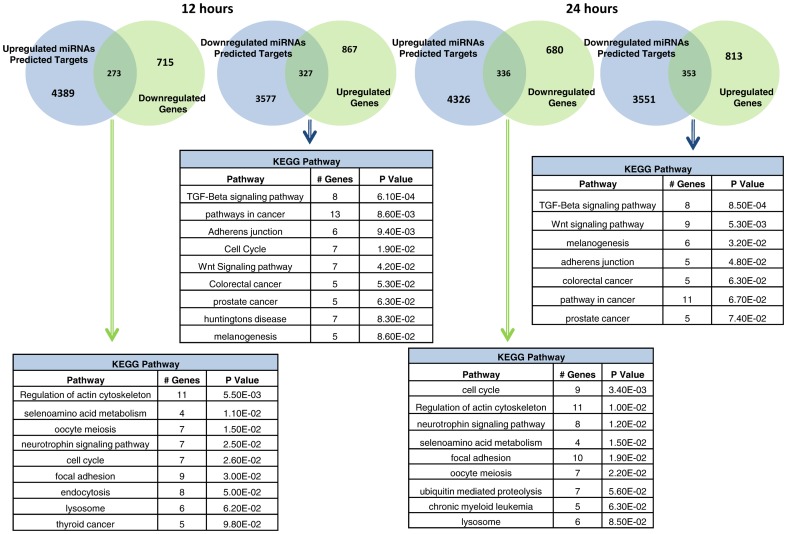
Venn diagram of predicted targets and the differentially regulated genes.

Using online tools for miRNA target prediction- miRecords or TargetScan Custom [18], [23], a total of ∼10,000 putative targets of the differentially regulated miRNAs were identified (**Data S7**). Subsequently, these genes obtained from target prediction were matched with those obtained from gene expression profiling and inverse correlations were identified (ie. miRNA is upregulated and the corresponding gene is downregulated and *vice versa*). About 2000 inverse correlations were predicted in total (**Figure 7** **, Data S8**). These listed targets were then used to conduct gene ontology and pathway analysis using DAVID software, which interestingly yielded results similar to the results obtained for the differentially expressed gene profile. This suggests that miRNA regulation of gene targets may have a profound effect on overall gene signature.

Interestingly, targets of the downregulated miRNAs were enriched in the TGF-β, Wnt and adherens junction pathway. Among the downregulated targets, cell cycle pathway is prominently downregulated. Other pathways like the regulation of actin cytoskeleton and selenoaminoacid metabolism were also suppressed (**Figure 7**).

## Discussion

In the past decade, CHIKV has presented itself with worrisome pathogenic effects urging the need to identify the key molecular players and fully dissect the events following CHIKV infection. The results presented here reveal the key transcripts and pathways involved in CHIKV pathogenesis in a human host cell line, HEK293T. To our knowledge this is a first comprehensive report of the host small RNA (miRNA and snoRNA) and mRNA signatures post CHIKV infection. Integration of miRNA target transcripts and mRNA profiles also helped us to identify complex miRNA∶mRNAinteractome networks involved.

Recent work has highlighted important role of miRNAs in host∶pathogen interactions. Accumulating evidences demonstrate differential regulation of both pathogen and host encoded miRNAs in response to infection that ensues in complex regulation of target genes [24]. However, there is no experimental or prediction based report yet on CHIKV encoded miRNAs. Moreover, the microarray platform based analysis used here is limited by the analysis of only known probesets as opposed to the deep sequencing platform where we can identify novel sequences. Thus, here we have analysed only host encoded miRNAs showing differential expression (>1.5 fold, p<0.05) post infection. We confirmed time dependent induction of specific miRNAs (miR-663, miR-638, miR-503 and miR-744) in response to CHIKV infection thus validating the microarray data. Interestingly, about 53% of the upregulated miRNAs and 45% of the downregulated miRNAs have been implicated in other viral infections, particularly Hepatitis C Virus, Hepatitis B Virus, Human Papilloma Virus and Human Immunodeficiency Virus 1 (**Figure 2** **, Data S4**). This may represent a general viral or host cell response common to these viral infections.

Interestingly, several members of miRNA clusters were also found to be induced. miR-17-92 cluster when upregulated, has been known to promote cell proliferation in human cells and has been proposed to be used as a propagation mechanism induced by various viruses [25]. On the other hand, miR-15/16 cluster (hsa-miR-15b and hsa-miR-16) promotes apoptosis by targeting BCL2 [26] and miR-23a cluster is known to induce caspase-dependant and independant apoptosis in HEK cell lines [27]. This can be a manifestation of the host response against the virus. Similarly, hsa-let-7e, hsa-miR-125a-3p and hsa-miR-99b are part of a highly conserved cluster known to regulate the number of hematopoietic stem cell, which could give rise to myeloid cells for defence against viral infection [28]. Interestingly, miR-23a cluster along with miR-106b cluster and miR-17-92 cluster are also known to regulate the TGF-β signaling pathway, which is highly induced post CHIKV infection (as shown above by our studies) [29], [30]. Overall, it would be important to study the specific roles of each of these miRNAs during CHIKV infection.

We found several snoRNAs too as being differentially expressed in response to CHIKV infection. This is particularly exciting in light of recent studies suggesting that several human C/D box snoRNAs may have miRNA like functions [31] or may act as precursors of miRNAs [32]. Strikingly, snoRNAs have been shown recently to be expressed from viral genomes with a putative important role to play in infection. For instance, C/D snoRNA has been shown to be expressed from the Epstein-Barr virus genome [33]. Our data shows induction of U3 and its close paralogs (U3-2, U3-2B, U3-3, U3-4), U44, U76 and U78 snoRNAs) while downregulation of HBII-85-26 C/D snoRNAs at 12 and 24 hrs post infection. Interestingly, U44, U76 and U78 are products of same cluster in human host gene GAS5 (Growth arrest-specific 5) [34], the expression of which has been linked to apoptosis in a number of studies [35]. At 24 hr post infection downregulation of various paralogs of HBII-85 snoRNA was seen (HBII-85-6, HBII-85-8, HBII-85-26, HBII-85-29) that have been implicated before in Prader-Willi syndrome [36].What role these snoRNAs may play would be interesting to find out.

We next identified the gene expression signature of CHIKV. Among the pathways seen to be induced post infection, notable were the TGF-β signaling pathway, endocytosis pathway and the adherens junction pathway. Induction of the endocytosis pathway can be explained due to greater uptake of viral particles while the adherens junction pathway could be the immediate host cell response to viral entry. Monitoring levels of genes in the adherens junction pathway for longer time periods may show the disruption of this pathway and breakdown of cell to cell contact, ultimately culminating in cell death. Previous studies showed that CHIKV mobilizes the apoptotic pathway of the host cell for proliferation [37] while another study showed that macrophages (induced as a defence mechanism by the host) ingest the apoptotic cells via mechanisms which involve TGF-β, Prostaglandin 2 and Platelet-activating factor Pathways [38]. Significant increase in the levels of the genes involved in the TGF-β signaling pathway (SMAD Dependent) could indicate a possible host cell response leading to uptake of the infected cell via macrophages. Upon application of a TGF-β receptor inhibitor to HEK293T cells, the CHIKV mediated cell death was much more as compared to cells not treated with the inhibitor. This may indicate that the TGF-β pathway somehow functions to abate the effect of CHIKV infection. However, these are preliminary results and the role of TGF-β pathway during CHIKV infection need to be further studied in detail. Interestingly, the pathway analysis of the miRNA target genes showing inverse correlations with the differentially regulated miRNAs showed similar pathways being overrepresented thus suggesting that the miRNAs may play a significant role in the regulation of genes involved in CHIKV pathogenesis and host response.

Similarly, the downregulated genes were largely represented by the cell cycle pathways genes or proteasome and lysosome pathways all indicating growth arrest or progression towards cell death caused by the viral infection. The downregulation of the genes involved in the regulation of the actin cytoskeleton may be induced by the virus to disrupt the cell membrane integrity and ease apoptosis.

The functional analysis of the upregulated genes point towards a general induction in the transcriptional machinery of the host cell, as shown by the induction of the genes involved in DNA-Binding and Transcription Regulator Activity. The upregulation of the genes in SMAD binding may be directly related to the induction of the SMAD dependent TGF-β Signaling Pathway.

We saw huge induction in the level of the chemokine receptor CXCR4 by both microarray and qRT-PCR post infection. This suggests that CHIKV similar to HIV may use CXCR4 receptor for viral entry in the epithelial cells [39]. This observation however needs functional confirmation.

## Conclusions

In summary, our study provides a platform to understand the dynamics of infection of Chikungunya Virus and its potential targets for the identification of therapeutic targets or biomarkers. This will aid in the development of anti-CHIKV drugs and diagnostic tools. Recently, Lanford and collaborators demonstrated that anti-miRNA molecules can be successfully used to treat chronic hepatitis C virus in chimpanzees [40]. The miRNA signature established in our studies could point towards potential miRNA targets which may be used for this purpose. Strong induction of the TGF-β pathway can also be further studied to find the susceptible targets which could be used for the abatement of CHIKV infection.

## Supporting Information

Data S1 **miRNA expression profiling data.** HEK293T cells were infected with CHIKV and miRNA expression profiling was done at 0, 12 and 24 hr post infection. The excel file sheets specifying miRNAs found to be more than 1.5 fold upregulated or downregulated at 12 and 24 hr post CHIKV infection as compared to 0 hr are shown along with the fold change values. The details of the profiling are given in Methods section.
(XLSX)

Data S2 **Gene expression profiling data.** HEK293T cells were infected with CHIKV and gene expression profiling was done at 0, 12 and 24 hr post infection. The excel file sheets specifying genes found to be more than 1.5 fold upregulated or downregulated at 12 and 24 hr post CHIKV infection as compared to 0 hr are shown along with the fold change values. The details of the profiling are given in Methods section. Hierarchial clustering of differentially expressed genes (>1.5 fold, p<0.05) in response to CHIKV infection at 12 and 24 hr as compared to 0 hr control in HEK293T cell line is also shown. A and B refer to the biological replicates.
(XLSX)

Data S3 **A list of primers used for detection of specific miRNA and mRNA transcripts.**
(DOCX)

Data S4 **List of CHIKV regulated miRNAs implicated in other virus pathogenesis or associated pathways based on literature survey.**
(XLSX)

Data S5 **A list of CHIKV specific differentially regulated host miRNAs.**
(DOC)

Data S6 **List of differentially regulated miRNA clusters.** (**A**) HEK293T Cells were infected with CHIKV and RNA harvested at 12 and 24 hr post infection. From the microarray data, the miRNA expressed as clusters were identified and listed. (**B**) Real-time PCR data showing differentially regulated miRNA clusters in response to CHIKV infection. The graphical data points represent mean+S.D of at least three independent experiments. (*P<0.05, **P<0.01). Error bars denote+SD.
(PPTX)

Data S7 **List of predicted targets of differentially regulated miRNAs using miRecords and Targetscan software.**
(XLSX)

Data S8 **List of predicted targets of differentially regulated miRNAs showing inverse correlations.**
(DOCX)
