# Supplementary material for: Combined miRNA and mRNA Signature Identifies Key Molecular Players and Pathways Involved in Chikungunya Virus Infection in Human Cells
Source: PLoS One. 2013 Nov 21;8(11):e79886. doi: 10.1371/journal.pone.0079886 (PMC3836776; doi:10.1371/journal.pone.0079886)
Supplement: Data S3 — A list of primers used for detection of specific miRNA and mRNA transcripts. (DOCX) [file pone.0079886.s003.docx]

|  | **Supplementary Data 3 : List of primers used** |
| --- | --- |
|  |  |
| **Primer Detail** | **Primer Sequence** |
| **Primers for Detection of Mature microRNA Level** | |
|  |  |
| MIR-25 Forward Primer | CGCCATTGCACTTGTCTCC |
| MIR-638 Forward Primer | TATAGGGATCGCGGGC |
| MIR-503 Forward Primer | CATAGCAGCGGGAACAG |
| MIR-10a Forward Primer | GCGTACCCTGTAGATCCG |
| MIR-663 Forward Primer | TAATAGGCGGGGCGC |
| MIR-17 Forward Primer | GCGCGCAAAGTGCTTACAGTG |
| MIR-19b Forward Primer | CGCGTGTGCAAATCCATGC |
| MIR-18a Forward Primer | GCGCGTAAGGTGCATCTAGTGC |
| MIR-20a Forward Primer | GCGCGTAAAGTGCTTATAGTGCAGG |
| MIR-744 Forward Primer | TATTGCGGGGCTAGGG |
| MIR-425 Forward Primer | GCCGAATGACACGATCACTCC |
| MIR-3175 Forward Primer | ATACGGGGAGAGAACGCAG |
| MIR-671-5p | ATAAGGAAGCCCTGGAGG |
| RNU6B Forward Primer | GCCCCTGCGCAAGGATGAC |
|  |  |
| MIR-191 RT Primer | GTCGTATCCAGTGCAGGGTCCGAGGTATTCGCACTGGATACGACCAGCTG |
| RNU6B RT Primer | GTCGTATCCAGTGCAGGGTCCGAGGTATTCGCACTGGATACGACAAAATATGGAAC |
| MIR-425 RT Primer | GTCGTATCCAGTGCAGGGTCCGAGGTATTCGCACTGGATACGACTCAACG |
| MIR-25 RT Primer | GTCGTATCCAGTGCAGGGTCCGAGGTATTCGCACTGGATACGACTCAGACC |
| MIR-744 RT Primer | GTCGTATCCAGTGCAGGGTCCGAGGTATTCGCACTGGATACGACTGCTGTTA |
| MIR-663 RT Primer | GTCGTATCCAGTGCAGGGTCCGAGGTATTCGCACTGGATACGACGCGGTCCC |
| MIR-638 RT Primer | GTCGTATCCAGTGCAGGGTCCGAGGTATTCGCACTGGATACGACAGGCCGCC |
| MIR-17 RT Primer | GTCGTATCCAGTGCAGGGTCCGAGGTATTCGCACTGGATACGACCTACCT |
| MIR-18a RT Primer | GTCGTATCCAGTGCAGGGTCCGAGGTATTCGCACTGGATACGACCTATCTG |
| MIR-20a RT Primer | GTCGTATCCAGTGCAGGGTCCGAGGTATTCGCACTGGATACGACCTATCTG |
| MIR-19b RT Primer | GTCGTATCCAGTGCAGGGTCCGAGGTATTCGCACTGGATACGACTCAGTT |
| MIR-3175 RT Primer | GTCGTATCCAGTGCAGGGTCCGAGGTATTCGCACTGGATACGACACGTCA |
| MIR-671-5p RT primer | GTCGTATCCAGTGCAGGGTCCGAGGTATTCGCACTGGATACGACCTCCAG |
| Stem-loop Reverse Universal | GTGCAGGGTCCGAGGT |
|  |  |
| **Primers for Detection of Gene Transcripts** | |
|  |  |
| JUN Forward Primer | GTGTGCACGAGTGGGAAGG |
| JUN  Reverse Primer | GATCGAATGTTAGGTCCATGCAG |
| HSPA8 Forward Primer | GAGGCACTGTCATCTGGTCTTG |
| HSPA8 Reverse Primer | ACTTCTCAGCTTCCTGGACCATAC |
| SMAD6 Forward Primer | GCTCTAGGAATGCAGACGCTG |
| SMAD6 Reverse Primer | CAACAGGCAGTCAGCACAGTC |
| CXCR4 Forward Primer | ATCCTGGCCTTCATCAGTCTGG |
| CXCR4 Reverse Primer | AACACAACCACCCACAAGTCATTG |
| SKIL Forward Primer | GCTCACTGAAACCTCCACCTC |
| SKIL  Reverse Primer | GGCTGGTGGATCACAAGG |
| ADRB1 Forward Primer | TCGATCATCGTGGCTCCC |
| ADRB1 Reverse Primer | GCCTCCATCCTTTCTCTTTGC |
| CDC23 Forward primer | TCCCTCCCATCTGCAACAG |
| CDC23 Reverse primer | AAGTCTGGACCCTGAGCTCAG |
| CDC27 Forward primer | AGAAGTTATGTTGTGGCCTTGG |
| CDC27 Reverse primer | AGGTACAACAGCAGCATGGTTC |
| YWHAE Forward Primer | GGAGGGACTTCGTTGTAATGG |
| YWHAE reverse primer | ACCACCTTCAACGCTAACCTG |
| PLK1 Forward Primer | TACATCGACGAGAAGCGGG |
| PLK1 Reverse Primer | TGGGAGTGAGGAGGGCAC |
