# Supplementary material for: Combined miRNA and mRNA Signature Identifies Key Molecular Players and Pathways Involved in Chikungunya Virus Infection in Human Cells
Source: PLoS One. 2013 Nov 21;8(11):e79886. doi: 10.1371/journal.pone.0079886 (PMC3836776; doi:10.1371/journal.pone.0079886)
Supplement: Data S5 — A list of CHIKV specific differentially regulated host miRNAs. (DOC) [file pone.0079886.s005.doc]

**Supplementary Data S5:** CHIKV specific differentially regulated host miRNAs.
