# Supplementary material for: Combined miRNA and mRNA Signature Identifies Key Molecular Players and Pathways Involved in Chikungunya Virus Infection in Human Cells
Source: PLoS One. 2013 Nov 21;8(11):e79886. doi: 10.1371/journal.pone.0079886 (PMC3836776; doi:10.1371/journal.pone.0079886)
Supplement: Data S8 — List of predicted targets of differentially regulated miRNAs showing inverse correlations. (DOCX) [file pone.0079886.s008.docx]

**Supplementary Data 8 : Inverse Correlation**

The gene expression profile data and the putative target list of the differentially expressed miRNAs were matched. The pairs showing inverse correlations are listed below.

**Downregulated miRNAs**

**12 hours post infection**

| **Downregulated miRNAs** | **Upregulated Genes** | | | |
| --- | --- | --- | --- | --- |
| **hsa-mir-1256** | PCMTD1 | ITPR1 | PCDH18 | SATB1 |
|  | BCL6 | KCNT2 | PCDH9 | TSPAN7 |
|  | CLEC2D | MGA | PHF17 | ZFP14 |
|  | DICER1 | OTUD4 | QKI | FBXW11 |
| **hsa-mir-1263** | PDZRN3 | ESRRG | PBX1 | UBE2W |
|  | PTPRF | FBXW11 | ST6GAL1 | ZIC3 |
|  | ZCCHC2 |  |  |  |
| **hsa-mir-126-5p** | CPEB2 | JPH1 | PCDH9 | PHTF2 |
|  | IKZF2 | KIAA1024 | PPM1B | PLCB4 |
|  | MEF2D | KIAA1033 | PTER | RALA |
|  | PLAG1 | KIAA1267 | SPAG9 | RBMS1 |
|  | CREBBP | MAK | STYX | RREB1 |
|  | FBXO3 | MYLIP | CLEC2D | USP6NL |
|  | INSIG1 | NCOA3 | PHF3 | ZFX |
|  | ITGB1 | PAPD5 |  |  |
| **hsa-mir-1272** | AMOT | GPM6A | TOP1 | SLC17A5 |
|  | ATRX | RBM18 | CREB5 | ZNF638 |
| **hsa-mir-1275** | CELSR2 | SOX4 | PHF17 | TCF7 |
|  | KCNJ11 | CAMTA1 | RTN4 | ZNF644 |
|  | PTCH1 |  |  |  |
| **hsa-mir-1280** | HOXB5 | PTBP2 | CDK6 | STK4 |
|  | NAV1 | ACVR1B | DIXDC1 | TNRC6A |
|  | GOLGA8A | BNIP3L | JUN | ZNF559 |
| **hsa-mir-1294** | ARG2 | ESRRG | MYO5A | PTBP2 |
|  | BCL11A | IKZF2 | NAB1 | ZBTB4 |
|  | BNIP3L | MYCL1 | PHTF2 | ZFX |
|  | CAMTA1 |  |  |  |
| **hsa-mir-1323** | ELAVL2 | ZBTB41 | DOCK4 | PGAP1 |
|  | KLF10 | AGGF1 | EDIL3 | PHACTR2 |
|  | AFF4 | ATXN3 | ENAH | PHF17 |
|  | CAMTA1 | IKZF2 | ESCO1 | PRDM8 |
|  | CPEB4 | PPP1R12A | GHR | RALA |
|  | HMGCS1 | RNF11 | GULP1 | RPS6KB1 |
|  | KCTD18 | UBE2W | INTS6 | SOX21 |
|  | PELI1 | ZIC3 | ITGB1 | SUGT1 |
|  | SIX4 | ZNF236 | JAG1 | SUZ12 |
|  | TOP1 | ZNRF1 | KCNT2 | TASP1 |
|  | GPM6A | BDP1 | KIAA1704 | TCF12 |
|  | SLC2A13 | C11orf30 | MAP3K2 | TMEM135 |
|  | AFTPH | C9orf5 | MCFD2 | TMEM65 |
|  | C14orf4 | CCNJ | MYCL1 | U2AF2 |
|  | KLF9 | CDC25A | OBFC2A | YIPF4 |
|  | OTUD4 | CP110 | OSTM1 | ZEB2 |
|  | PTBP2 | DEK | PANK1 | ZNF398 |
| **hsa-mir-200b-5p** | CAMTA1 | NAV1 | ZFX | GALK2 |
|  | FLRT3 | CNOT8 | ANK2 | HECTD2 |
|  | PELI1 | GULP1 | DIXDC1 | PRPF4B |
|  | SUZ12 | IKZF2 | DPP4 | RNF11 |
|  | VCAN | PPP1R12A | ENAH | SATB1 |
|  | VLDLR | TCERG1 |  |  |
| **hsa-mir-202** | DICER1 | IPO5 | GK5 | ONECUT2 |
|  | SLCO4C1 | GHR | ERO1L | YAF2 |
|  | NIPA1 | YPEL2 | C2orf3 | STK4 |
|  | ZNF823 |  |  |  |
| **hsa-mir-202-5p** | FAM46C | APPBP2 | SPRED1 | RBMS1 |
|  | TCF12 | CREBBP | YAF2 | RPS6KB1 |
|  | RNF11 | ENAH | CTDSPL2 | SIX4 |
|  | ANP32E | FAM76B | INTS6 | TSPAN13 |
|  | BCL11A | IQGAP1 | MPPED2 | ZNF148 |
|  | PGAP1 | ROBO2 | OXR1 | ZNF800 |
|  | ACVR1 | SNX16 |  |  |
| **hsa-mir-221-5p** | DDX17 | TNRC6A | MYO1E | RPS6KB1 |
|  | NCOA3 | EDEM3 | NFKBIZ | THRB |
|  | ONECUT2 | HIVEP1 | PCDH9 | ZCCHC2 |
|  | OXR1 | MGA |  |  |
| **hsa-mir-3128** | ACVR1 | EML5 | ROD1 | C9orf5 |
|  | PHACTR2 | MBNL2 | SLC2A13 | ESRRG |
|  | ABCA1 | NBEA | SNX13 | TMEM135 |
|  | CDC27 | NDST1 | TBP | UBE2V1 |
|  | CSNK1G1 |  |  |  |
| **hsa-mir-32** | SOX4 | POLK | ATRX | CDK6 |
|  | GOLGA3 | ATRX | PCMTD1 | FXR1 |
|  | ITPR1 | TMEM188 | ROBO2 | GIT2 |
|  | PPP1R12A | HERPUD2 | FOXN2 | SOCS5 |
|  | DMXL1 | RAB18 | NRIP1 | DOCK9 |
|  | ATXN3 | CPEB2 | CREB1 | NEFL |
|  | PHTF2 | GOLGA8A | GAP43 | MEF2D |
|  | LATS2 | SPRYD4 | UBE2W | SMAD6 |
|  | CPEB4 | QKI | MYO5A | APPL1 |
|  | OTUD4 | SOCS5 | ESRRG | BCL11A |
|  | INSIG1 | EDEM3 |  |  |
| **hsa-mir-3201** | JUN | QKI | RNF11 | TOP1 |
|  | MYLK | ROBO2 | C14orf4 | CPEB4 |
|  | CAMTA1 | PDK1 | SOX4 | MYCL1 |
|  | BCL6 | CELSR2 | DLL1 | CTDSPL2 |
| **hsa-mir-320a** | ARNTL | CPEB4 | PPP1R8 | DOCK4 |
|  | CREB5 | ENAH | PRPF4B | ESRRG |
|  | EIF2AK3 | ESCO1 | RAB18 | FAM76B |
|  | ONECUT2 | FLRT3 | RSF1 | HDHD2 |
|  | THRB | GOLGA3 | ST6GALNAC3 | KLF9 |
|  | AFF4 | GPM6A | TFDP2 | NCOA3 |
|  | BDP1 | IRF2 | TMEM108 | PHTF2 |
|  | ELAVL2 | JPH1 | TMEM135 | RBMS1 |
|  | FAM84B | MGA | ZIC3 | SNX13 |
|  | HECTD2 | MIER1 | ABI2 | STK4 |
|  | PPM1B | NDST1 | ATRX | TAOK3 |
|  | TNRC6A | OTUD4 | CCDC6 | TMEM106B |
|  | UBE2W | PCDH18 | CHD7 | TOP1 |
|  | ZNF423 | PCGF5 | CPEB2 | UBE2V1 |
|  | ADK | PDZRN3 | CTTNBP2NL | ZNF148 |
|  | ATP11B | PLAG1 |  |  |
| **hsa-mir-320b** | ARNTL | CPEB4 | PPP1R8 | DOCK4 |
|  | CREB5 | ENAH | PRPF4B | ESRRG |
|  | EIF2AK3 | ESCO1 | RAB18 | FAM76B |
|  | ONECUT2 | FLRT3 | RSF1 | HDHD2 |
|  | THRB | GOLGA3 | ST6GALNAC3 | KLF9 |
|  | AFF4 | GPM6A | TFDP2 | NCOA3 |
|  | BDP1 | IRF2 | TMEM108 | PHTF2 |
|  | ELAVL2 | JPH1 | TMEM135 | RBMS1 |
|  | FAM84B | MGA | ZIC3 | SNX13 |
|  | HECTD2 | MIER1 | ABI2 | STK4 |
|  | PPM1B | NDST1 | ATRX | TAOK3 |
|  | TNRC6A | OTUD4 | CCDC6 | TMEM106B |
|  | UBE2W | PCDH18 | CHD7 | TOP1 |
|  | ZNF423 | PCGF5 | CPEB2 | UBE2V1 |
|  | ADK | PDZRN3 | CTTNBP2NL | ZNF148 |
|  | ATP11B | PLAG1 |  |  |
| **hsa-mir-320c** | ARNTL | CPEB4 | PPP1R8 | DOCK4 |
|  | CREB5 | ENAH | PRPF4B | ESRRG |
|  | EIF2AK3 | ESCO1 | RAB18 | FAM76B |
|  | ONECUT2 | FLRT3 | RSF1 | HDHD2 |
|  | THRB | GOLGA3 | ST6GALNAC3 | KLF9 |
|  | AFF4 | GPM6A | TFDP2 | NCOA3 |
|  | BDP1 | IRF2 | TMEM108 | PHTF2 |
|  | ELAVL2 | JPH1 | TMEM135 | RBMS1 |
|  | FAM84B | MGA | ZIC3 | SNX13 |
|  | HECTD2 | MIER1 | ABI2 | STK4 |
|  | PPM1B | NDST1 | ATRX | TAOK3 |
|  | TNRC6A | OTUD4 | CCDC6 | TMEM106B |
|  | UBE2W | PCDH18 | CHD7 | TOP1 |
|  | ZNF423 | PCGF5 | CPEB2 | UBE2V1 |
|  | ADK | PDZRN3 | CTTNBP2NL | ZNF148 |
|  | ATP11B | PLAG1 |  |  |
| **hsa-mir-335** | UBE2H | UBE2H | FCHSD2 | JAG1 |
|  | KLHL28 | FAM84B | DOCK4 |  |
| **hsa-mir-450b-5p** | C14orf4 | PPP1R12A | GPM6A | BCL6 |
|  | CTDSPL2 | RPS6KB1 | IKZF2 | CHD7 |
|  | CXorf23 | SP8 | JAG1 | CLEC2D |
|  | EIF2C3 | ZFX | LHX8 | EIF4G3 |
|  | ESRRG | SOX4 | MAP1B | ETV1 |
|  | ITGA6 | CAMTA1 | PLAG1 | KLHL28 |
|  | OXR1 | ABCA1 | QKI | RBMS1 |
|  | PAPD5 | BICD2 | SPOCK3 | SLMAP |
|  | PAX6 | CCDC6 | TNRC6A | SPIRE1 |
|  | PHF17 | FAM70A | USP25 | TAOK3 |
| **hsa-mir-451** | ZNF644 | MEX3C | C11orf30 |  |
| **hsa-mir-455-3p** | EIF4E | PCDH9 | GPC4 | ZFX |
|  | EIF4G3 | BMPR1A | PRPF4B | ZNF423 |
|  | DLL1 | ESRRG |  |  |
| **hsa-miR-548a-3p** | LMBR1 | PPM1B | PROS1 | NEK1 |
|  | USP31 | NUDT12 | ANKRD34B | NNT |
|  | ZBTB4 | KCTD10 | NARG2 | ZNF184 |
|  | DOCK9 | PGAP1 | CREB5 | ZXDB |
|  | CEP57 | BRWD1 | CACYBP | ZFP161 |
|  | KIAA0895 | FAM134B | ENAH | PPM1D |
|  | ZNF562 | DIXDC1 | AFF3 | SSR1 |
|  | KLHL28 | GJC1 | COL13A1 | PDK1 |
|  | PAQR5 | HMGCS1 | RAD51AP1 | PDK4 |
|  | SHPRH | GPC4 | MAP3K2 | CPNE3 |
|  | INSIG1 | ANK2 | DDX17 | ARHGAP29 |
|  | NAPEPLD | KLF9 | TSPAN2 | ONECUT2 |
|  | QKI | SMAD2 | ABI2 | ATXN3 |
|  | ZBTB41 | LEPR | NAB1 | SOCS6 |
|  | CREB3L2 | MYO5A |  |  |
| **hsa-mir-548u** | ELAVL2 | GNPTAB | AFF3 | ONECUT2 |
|  | MTHFD1L | HIVEP1 | APP | PGAP1 |
|  | PAPD5 | HOOK3 | CHD9 | PHF17 |
|  | UBE2W | HOXB5 | CPEB2 | STRBP |
|  | KIAA1033 | MYO10 | DPP4 | TRIM2 |
|  | ZEB2 | NRXN3 | FAM84B | USP46 |
|  | AFTPH | PAWR | ITPR1 | ZFYVE16 |
|  | BCL11A | TFDP2 | MOSPD2 | ZNF148 |
|  | FYCO1 |  |  |  |
| **hsa-mir-548x** | ELAVL2 | GNPTAB | AFF3 | ONECUT2 |
|  | MTHFD1L | HIVEP1 | APP | PGAP1 |
|  | PAPD5 | HOOK3 | CHD9 | PHF17 |
|  | UBE2W | HOXB5 | CPEB2 | STRBP |
|  | KIAA1033 | MYO10 | DPP4 | TRIM2 |
|  | ZEB2 | NRXN3 | FAM84B | USP46 |
|  | AFTPH | PAWR | ITPR1 | ZFYVE16 |
|  | BCL11A | TFDP2 | MOSPD2 | ZNF148 |
|  | FYCO1 |  |  |  |
| **hsa-mir-603** | ZCCHC2 | MPP7 | DNAJC21 | PLAG1 |
|  | PMEPA1 | FREM2 | CREB5 | TOP1 |
|  | AFF4 | C9orf5 | THRB | ZNF264 |
|  | PDS5B | PCGF5 | ATM | PRPF4B |
|  | EEF2K | D4S234E | UBE2K | CPNE3 |
|  | C18orf19 | ZNF197 | SYNCRIP | IQGAP1 |
|  | AMOT | CDK6 | FBXW11 |  |
| **hsa-mir-606** | C10orf140 | BRWD1 | NARG2 | HOXB3 |
|  | GNPTAB | ONECUT2 | PAPD5 | SMAD2 |
| **hsa-mir-628-5p** | SNX13 | FKBP1A | PAPD5 | PRPF4B |
|  | ACVR1B | FOXP1 | PCGF5 | PTCH1 |
|  | ANK2 | GLIS2 | UBE2W | ZNF148 |
|  | APPBP2 | IKZF2 | CSNK1G1 |  |
| **hsa-mir-671-5p** | USP46 | EDEM3 | ROBO2 | GPM6A |
|  | VGLL3 | ZNF585A | ONECUT2 | CCDC6 |
| **hsa-mir-720** | CAMTA1 |  |  |  |

**24 hours post infection**

| **Downregulated miRNAs** | **Upregulated Genes** | | | |
| --- | --- | --- | --- | --- |
| **hsa-miR-1184** | MEF2D | IGF2BP2 | FAM76B | PTBP2 |
|  | CTDSPL2 | PRPF4B | FOXN2 | SIPA1L2 |
|  | BCL6 | SOX4 | GHR | SNX13 |
|  | FXR1 | SP8 | KLF9 | TBP |
| **hsa-mir-1246** | ACVR1 | ZIC3 | QKI | MPP5 |
|  | PLAG1 | BCL11A | CTTNBP2 | NDST1 |
|  | ABCA1 | CPEB2 | GPM6A | RSRC2 |
|  | MBNL2 | EIF2AK3 | HDHD2 | TRIM2 |
| **hsa-mir-1256** | PCMTD1 | KCNT2 | PCDH9 | TSPAN7 |
|  | BCL6 | MGA | PHF17 | ZFP14 |
|  | CLEC2D | OTUD4 | QKI | FBXW11 |
|  | ITPR1 | PCDH18 | SATB1 |  |
| **hsa-mir-1260b** | FAM46C | FAM76B |  |  |
| **hsa-mir-1263** | PDZRN3 | ZCCHC2 | FBXW11 | UBE2W |
|  | PTPRF | ESRRG | ST6GAL1 | ZIC3 |
| **hsa-mir-126-5p** | CPEB2 | INSIG1 | PAPD5 | PHTF2 |
|  | IKZF2 | JPH1 | PCDH9 | PLCB4 |
|  | MEF2D | KIAA1024 | PPM1B | RALA |
|  | PLAG1 | KIAA1267 | PTER | RBMS1 |
|  | CHCHD4 | MAK | STYX | RREB1 |
|  | CREBBP | MYLIP | CLEC2D | USP6NL |
|  | FBXO3 | NCOA3 | PHF3 | ZFX |
| **hsa-mir-1272** | AMOT | RBM18 | SLC17A5 | ZNF638 |
|  | GPM6A | CREB5 |  |  |
| **hsa-mir-1274a** | FAM126B | BCL6 | NIPA1 | MAPT |
|  | PHF17 | CPEB4 | RAPGEF5 | SNX13 |
|  | SNX24 | CSNK1G1 | ARHGAP29 | ZNRF1 |
|  | VDR | FBXL20 | CREB1 |  |
| **hsa-mir-1274b** | ONECUT2 | C11orf30 | MCL1 | RHOBTB1 |
|  | ZNF148 | DLG3 | MEF2D | ZBTB4 |
|  | BCL6 | ENAH | MGA | ZNF2 |
|  | ZBED4 | ESRRG | NRIP1 | BRWD1 |
|  | ATXN3 |  |  |  |
| **hsa-mir-1275** | CELSR2 | PTCH1 | CAMTA1 | RTN4 |
|  | KCNJ11 | SOX4 | PHF17 | TCF7 |
| **hsa-mir-1280** | NAV1 | ACVR1B | DIXDC1 | STK4 |
|  | GOLGA8A | BNIP3L | JUN | TNRC6A |
|  | PTBP2 |  |  |  |
| **hsa-mir-1294** | MYCL1 | ARG2 | ESRRG | PTBP2 |
|  | CAMTA1 | BCL11A | IKZF2 | ZBTB4 |
|  | MYO5A | BNIP3L | PHTF2 | ZFX |
|  | NAB1 |  |  |  |
| **hsa-mir-1323** | ELAVL2 | ZBTB41 | ENAH | PHF17 |
|  | KLF10 | ATXN3 | ESCO1 | PRDM8 |
|  | AFF4 | IKZF2 | GHR | RALA |
|  | CAMTA1 | PPP1R12A | GULP1 | RPS6KB1 |
|  | CPEB4 | RNF11 | INTS6 | SOX21 |
|  | HMGCS1 | UBE2W | JAG1 | SUGT1 |
|  | KCTD18 | ZIC3 | KCNT2 | SUZ12 |
|  | PELI1 | ZNF236 | KIAA1704 | TASP1 |
|  | SIX4 | ZNRF1 | MAP3K2 | TCF12 |
|  | GPM6A | BDP1 | MCFD2 | TMEM135 |
|  | SLC2A13 | C11orf30 | MYCL1 | TMEM30A |
|  | AFTPH | C9orf5 | OBFC2A | TMEM65 |
|  | C14orf4 | CCNJ | OSTM1 | U2AF2 |
|  | KLF9 | CDC25A | PANK1 | YIPF4 |
|  | OTUD4 | DOCK4 | PGAP1 | ZEB2 |
|  | PTBP2 | EDIL3 | PHACTR2 | ZNF398 |
| **hsa-mir-144-5p** | ETV1 | PCDH9 | RPS6KB1 | TMEM161B |
|  | IGF2BP2 | PPP1R12A | SH3RF1 | ZFP1 |
|  | MBNL2 | RNF11 |  |  |
| **hsa-mir-151-3p** | PDS5B |  |  |  |
| **hsa-mir-200b-5p** | CAMTA1 | VLDLR | ZFX | HECTD2 |
|  | FLRT3 | NAV1 | ANK2 | PRPF4B |
|  | HNRPDL | CNOT8 | DIXDC1 | RNF11 |
|  | PELI1 | GULP1 | DPP4 | SATB1 |
|  | SUZ12 | IKZF2 | ENAH | TBL1XR1 |
|  | VCAN | PPP1R12A | GALK2 |  |
| **hsa-mir-202** | SLCO4C1 | IPO5 | GK5 | ONECUT2 |
|  | NIPA1 | GHR | ERO1L | YAF2 |
|  | ZNF823 | YPEL2 | C2orf3 | STK4 |
| **hsa-mir-202-5p** | FAM46C | APPBP2 | SPRED1 | RBMS1 |
|  | TCF12 | CREBBP | YAF2 | RPS6KB1 |
|  | RNF11 | ENAH | CTDSPL2 | SIX4 |
|  | ANP32E | FAM76B | INTS6 | TSPAN13 |
|  | BCL11A | ROBO2 | MPPED2 | ZNF148 |
|  | PGAP1 | SNX16 | OXR1 | ZNF800 |
|  | ACVR1 |  |  |  |
| **hsa-mir-2054** | BICD2 | NAB1 | CHD9 | NRIP1 |
|  | EDEM3 | PRPF4B | COL13A1 | ONECUT2 |
|  | KLF9 | TMEM161B | CPEB2 | OSBPL8 |
|  | CPEB4 | TRIM2 | ERO1L | PPP1R12A |
|  | EDNRA | ARHGEF3 | ESCO1 | RFFL |
|  | TNRC6A | BRWD1 | HDHD2 | TMEFF1 |
|  | ABCA1 | BTF3L4 | IGFBP7 | TMEM108 |
|  | KIAA1712 | CALML4 | MCL1 | USP32 |
|  | MKX | CHD7 | NCOA3 |  |
| **hsa-mir-221-5p** | NCOA3 | EDEM3 | MYO1E | RPS6KB1 |
|  | ONECUT2 | HIVEP1 | NFKBIZ | THRB |
|  | OXR1 | MGA | PCDH9 | ZCCHC2 |
|  | TNRC6A |  |  |  |
| **hsa-mir-3128** | ACVR1 | EML5 | ROD1 | C9orf5 |
|  | PHACTR2 | MBNL2 | SLC2A13 | ESRRG |
|  | ABCA1 | NBEA | SNX13 | TMEM135 |
|  | CSNK1G1 | NDST1 | TBP | UBE2V1 |
| **hsa-mir-32** | SOX4 | INSIG1 | EDEM3 | ESRRG |
|  | GOLGA3 | BCL11A | TBL1XR1 | FXR1 |
|  | ITPR1 | POLK | PCMTD1 | GIT2 |
|  | PPP1R12A | TMEM188 | ROBO2 | SOCS5 |
|  | DMXL1 | HERPUD2 | FOXN2 | DOCK9 |
|  | ATXN3 | RAB18 | NRIP1 | NEFL |
|  | PHTF2 | CPEB2 | CREB1 | MEF2D |
|  | LATS2 | GOLGA8A | GAP43 | SMAD6 |
|  | CPEB4 | QKI | UBE2W | APPL1 |
|  | OTUD4 | SOCS5 | MYO5A |  |
| **hsa-mir-3201** | JUN | ROBO2 | SOX4 | CTDSPL2 |
|  | MYLK | PDK1 | DLL1 | C14orf4 |
|  | CAMTA1 | CELSR2 | CPEB4 | QKI |
|  | BCL6 | RNF11 | MYCL1 |  |
| **hsa-mir-320a** | ARNTL | CPEB4 | PCGF5 | CPEB2 |
|  | CREB5 | ENAH | PDZRN3 | CTTNBP2NL |
|  | EIF2AK3 | ESCO1 | PLAG1 | DOCK4 |
|  | ONECUT2 | FLRT3 | PPP1R8 | ESRRG |
|  | THRB | GOLGA3 | PRPF4B | FAM76B |
|  | AFF4 | GPM6A | RAB18 | HDHD2 |
|  | BDP1 | IRF2 | RSF1 | KLF9 |
|  | ELAVL2 | JPH1 | ST6GALNAC3 | NCOA3 |
|  | FAM84B | MCL1 | TFDP2 | PHTF2 |
|  | HECTD2 | MGA | TMEM108 | RBMS1 |
|  | PPM1B | MIER1 | TMEM135 | SNX13 |
|  | TNRC6A | NDST1 | ZIC3 | STK4 |
|  | UBE2W | OTUD4 | ABI2 | TAOK3 |
|  | ZNF423 | PCDH18 | CCDC6 | TBL1XR1 |
|  | ADK | CHD7 | ZNF148 | UBE2V1 |
|  | ATP11B |  |  |  |
| **hsa-miR-320b** | ARNTL | CPEB4 | PCGF5 | CPEB2 |
|  | CREB5 | ENAH | PDZRN3 | CTTNBP2NL |
|  | EIF2AK3 | ESCO1 | PLAG1 | DOCK4 |
|  | ONECUT2 | FLRT3 | PPP1R8 | ESRRG |
|  | THRB | GOLGA3 | PRPF4B | FAM76B |
|  | AFF4 | GPM6A | RAB18 | HDHD2 |
|  | BDP1 | IRF2 | RSF1 | KLF9 |
|  | ELAVL2 | JPH1 | ST6GALNAC3 | NCOA3 |
|  | FAM84B | MCL1 | TFDP2 | PHTF2 |
|  | HECTD2 | MGA | TMEM108 | RBMS1 |
|  | PPM1B | MIER1 | TMEM135 | SNX13 |
|  | TNRC6A | NDST1 | ZIC3 | STK4 |
|  | UBE2W | OTUD4 | ABI2 | TAOK3 |
|  | ZNF423 | PCDH18 | CCDC6 | TBL1XR1 |
|  | ADK | CHD7 | ZNF148 | UBE2V1 |
|  | ATP11B |  |  |  |
| **hsa-miR-320c** | ARNTL | CPEB4 | PCGF5 | CPEB2 |
|  | CREB5 | ENAH | PDZRN3 | CTTNBP2NL |
|  | EIF2AK3 | ESCO1 | PLAG1 | DOCK4 |
|  | ONECUT2 | FLRT3 | PPP1R8 | ESRRG |
|  | THRB | GOLGA3 | PRPF4B | FAM76B |
|  | AFF4 | GPM6A | RAB18 | HDHD2 |
|  | BDP1 | IRF2 | RSF1 | KLF9 |
|  | ELAVL2 | JPH1 | ST6GALNAC3 | NCOA3 |
|  | FAM84B | MCL1 | TFDP2 | PHTF2 |
|  | HECTD2 | MGA | TMEM108 | RBMS1 |
|  | PPM1B | MIER1 | TMEM135 | SNX13 |
|  | TNRC6A | NDST1 | ZIC3 | STK4 |
|  | UBE2W | OTUD4 | ABI2 | TAOK3 |
|  | ZNF423 | PCDH18 | CCDC6 | TBL1XR1 |
|  | ADK | CHD7 | ZNF148 | UBE2V1 |
|  | ATP11B |  |  |  |
| **hsa-miR-320d** | ARNTL | CPEB4 | PCGF5 | CPEB2 |
|  | CREB5 | ENAH | PDZRN3 | CTTNBP2NL |
|  | EIF2AK3 | ESCO1 | PLAG1 | DOCK4 |
|  | ONECUT2 | FLRT3 | PPP1R8 | ESRRG |
|  | THRB | GOLGA3 | PRPF4B | FAM76B |
|  | AFF4 | GPM6A | RAB18 | HDHD2 |
|  | BDP1 | IRF2 | RSF1 | KLF9 |
|  | ELAVL2 | JPH1 | ST6GALNAC3 | NCOA3 |
|  | FAM84B | MCL1 | TFDP2 | PHTF2 |
|  | HECTD2 | MGA | TMEM108 | RBMS1 |
|  | PPM1B | MIER1 | TMEM135 | SNX13 |
|  | TNRC6A | NDST1 | ZIC3 | STK4 |
|  | UBE2W | OTUD4 | ABI2 | TAOK3 |
|  | ZNF423 | PCDH18 | CCDC6 | TBL1XR1 |
|  | ADK | CHD7 | ZNF148 | UBE2V1 |
|  | ATP11B |  |  |  |
| **hsa-mir-335** | UBE2H | FAM84B | DOCK4 | JAG1 |
|  | KLHL28 | FCHSD2 |  |  |
| **hsa-mir-378** | C9orf5 | C5orf41 |  |  |
| **hsa-mir-378c** | C9orf5 | C5orf41 |  |  |
| **hsa-mir-422a** | C9orf5 | C5orf41 |  |  |
| **hsa-mir-450b-5p** | ABCA1 | EIF2C3 | LHX8 | SIAH1 |
|  | BCL6 | EIF4G3 | OXR1 | SOX4 |
|  | BICD2 | ESRRG | PAPD5 | SP8 |
|  | C14orf4 | ETV1 | PAX6 | SPIRE1 |
|  | CAMTA1 | FAM70A | PHF17 | SPOCK3 |
|  | CCDC6 | GPM6A | PLAG1 | TAOK3 |
|  | CHD7 | IKZF2 | PPP1R12A | TBL1XR1 |
|  | CLEC2D | ITGA6 | QKI | TNRC6A |
|  | CTDSPL2 | JAG1 | RBMS1 | USP25 |
|  | CXorf23 | KLHL28 | RPS6KB1 | ZFX |
| **hsa-mir-451** | MEX3C | C11orf30 |  |  |
| **hsa-mir-455-3p** | EIF4E | PCDH9 | GPC4 | ZFX |
|  | EIF4G3 | BMPR1A | PRPF4B | ZNF423 |
|  | DLL1 | ESRRG |  |  |
| **hsa-miR-548a-3p** | LMBR1 | PPM1B | SMAD2 | ABI2 |
|  | USP31 | NUDT12 | LEPR | NAB1 |
|  | ZBTB4 | KCTD10 | MYO5A | NNT |
|  | DOCK9 | TBL1XR1 | PROS1 | ZNF184 |
|  | CEP57 | PGAP1 | ANKRD34B | ZXDB |
|  | KIAA0895 | BRWD1 | NARG2 | ZFP161 |
|  | ZNF562 | FAM134B | CREB5 | PPM1D |
|  | KLHL28 | DIXDC1 | CACYBP | SSR1 |
|  | PAQR5 | CDC42SE2 | ENAH | PDK1 |
|  | SHPRH | GJC1 | AFF3 | PDK4 |
|  | INSIG1 | HMGCS1 | COL13A1 | ARHGAP29 |
|  | NAPEPLD | GPC4 | RAD51AP1 | ONECUT2 |
|  | QKI | ANK2 | MAP3K2 | ATXN3 |
|  | ZBTB41 | KLF9 | TSPAN2 | SOCS6 |
|  | CREB3L2 |  |  |  |
| **hsa-mir-548u** | ELAVL2 | GNPTAB | APP | ONECUT2 |
|  | MTHFD1L | HIVEP1 | CHD9 | PGAP1 |
|  | PAPD5 | HOOK3 | CPEB2 | PHF17 |
|  | UBE2W | MYO10 | DPP4 | STRBP |
|  | ZEB2 | NRXN3 | FAM84B | TRIM2 |
|  | AFTPH | PAWR | ITPR1 | USP46 |
|  | BCL11A | TFDP2 | MCL1 | ZFYVE16 |
|  | FYCO1 | AFF3 | MOSPD2 | ZNF148 |
| **hsa-mir-548x** | ELAVL2 | GNPTAB | APP | ONECUT2 |
|  | MTHFD1L | HIVEP1 | CHD9 | PGAP1 |
|  | PAPD5 | HOOK3 | CPEB2 | PHF17 |
|  | UBE2W | MYO10 | DPP4 | STRBP |
|  | ZEB2 | NRXN3 | FAM84B | TRIM2 |
|  | AFTPH | PAWR | ITPR1 | USP46 |
|  | BCL11A | TFDP2 | MCL1 | ZFYVE16 |
|  | FYCO1 | AFF3 | MOSPD2 | ZNF148 |
| **hsa-mir-603** | ZCCHC2 | C18orf19 | D4S234E | UBE2K |
|  | PMEPA1 | AMOT | ZNF197 | SYNCRIP |
|  | AGPAT4 | MPP7 | DNAJC21 | FBXW11 |
|  | AFF4 | FREM2 | CREB5 | PLAG1 |
|  | PDS5B | C9orf5 | THRB | ZNF264 |
|  | EEF2K | PCGF5 | ATM | PRPF4B |
| **hsa-mir-606** | C10orf140 | HNRPDL | NARG2 | HOXB3 |
|  | GNPTAB | ONECUT2 | PAPD5 | SMAD2 |
|  | BRWD1 |  |  |  |
| **hsa-mir-628-5p** | SNX13 | FKBP1A | PAPD5 | PRPF4B |
|  | ACVR1B | FOXP1 | PCGF5 | PTCH1 |
|  | ANK2 | GLIS2 | UBE2W | ZNF148 |
|  | APPBP2 | IKZF2 | CSNK1G1 |  |
| **hsa-mir-671-5p** | USP46 | EDEM3 | ROBO2 | GPM6A |
|  | VGLL3 | ZNF585A | ONECUT2 | CCDC6 |
| **hsa-mir-720** | CAMTA1 |  |  |  |
| **hsa-mir-93** | CAMTA1 | C1orf63 | NIPA1 | GAB1 |
|  | ZNF148 | ZBTB4 | MBNL2 | OSBPL8 |
|  | ETV1 | MCL1 | FBXW11 | YPEL2 |
|  | OTUD4 | ENPP5 | FBXW11 | INTS6 |
|  | ZNF800 | CCNJ | VLDLR | BICD2 |
|  | NAPEPLD | PRDM8 | PLAG1 | TBC1D8B |
|  | UBE2W | FAM126B | PTPN3 | KLHL28 |
|  | MCL1 | C5orf41 | ELK3 | FAM46C |
|  | UBE2W | MUTED | ABCA1 | MAP3K2 |
|  | RSRC2 | RSRC2 | CCNG2 | ZBTB6 |
|  | BCL11A | NCOA3 | LHX8 | TXNIP |
|  | USP46 | SLC16A9 | EDNRB | CEP57 |
|  | ARHGEF3 | RNF128 | APP | TNRC6A |
|  | PHTF2 | GAB1 | FLT1 | FBXW11 |
|  | C11orf30 | RNF128 |  |  |

**Upregulated miRNAs**

**12 hours post infection**

| **Upregulated miRNAs** | **Downregulated Genes** | | | |
| --- | --- | --- | --- | --- |
| **hsa-let-7a** | AP1S1 | BZW2 | DLST | RBM38 |
|  | BIN3 | CAP1 | IGF2BP1 | RDX |
|  | BZW1 | CHD7 | MAPK6 | RPUSD3 |
|  | STX3 | SURF4 | WAPAL | SLC25A24 |
|  | ZNF275 |  |  |  |
| **hsa-miR-103** | BLMH | KIF23 | CAB39 | EIF5 |
|  | BSDC1 | PCGF5 | PHF20L1 | PI4KB |
|  | SMARCE1 |  |  |  |
| **hsa-mir-106a** | CCNG2 | NPLOC4 | SUV420H1 | MCL1 |
|  | CRK | OCRL | WAC | ZNF148 |
|  | SERP1 |  |  |  |
| **hsa-mir-107** | BLMH | EIF5 | PHF20L1 | TWF1 |
|  | BSDC1 | KIF23 | PI4KB | SMARCE1 |
|  | CAB39 | PCGF5 |  |  |
| **hsa-mir-10a** | CADM1 | CAMK2G | TMEM183A | USP46 |
| **hsa-mir-1202** | ARID4B | MTA2 | USP8 | BRMS1 |
|  | SIAH1 | TCEAL8 | RALY | DSP |
|  | CNOT3 | MLEC | CLASP2 |  |
| **hsa-mir-1207-5p** | CBX5 | RBM14 | IMPDH1 | CCDC86 |
|  | SH3GLB2 | MCRS1 | TAF9B | KLHDC3 |
|  | DUSP3 | C20orf29 | EPHX1 | AK1 |
|  | PBX1 | CNOT3 | MCL1 | USP46 |
|  | PRPF4 | GNB2 | CDK2AP2 | LOXL1 |
|  | C20orf27 | OSBPL8 |  |  |
| **hsa-mir-1224-5p** | UBAP2L | DDX3X | TAF9B | MLEC |
|  | ZNF275 | TSTA3 | CELF2 | SP1 |
| **hsa-mir-1225-5p** | CDC14B | SNRPA | TSPAN3 | CLSPN |
|  | PABPC4 | CSK |  |  |
| **hsa-mir-1231** | SNRPA | ARID4B | CLTC | ZNF148 |
|  | SEC23IP | NACC2 |  |  |
| **hsa-mir-1290** | VPS45 | ZNF24 | PARVA | TAF9B |
|  | PICALM | STK3 | PPP1CB | HAUS6 |
|  | SDHC | HNRPDL | CBX5 | ABT1 |
|  | CDC27 | SEC23IP | NACC2 | TNPO2 |
|  | CIZ1 |  |  |  |
| **hsa-mir-140-3p** | APLP2 | CAPRIN1 | LARP1 | ARF5 |
|  | YWHAZ | HYOU1 | PI4KB | PPP1CB |
| **hsa-mir-151-5p** | WIPI2 |  |  |  |
| **hsa-mir-152** | AKAP1 | CHD7 | TBL1XR1 | PPP1CB |
|  | ARL6IP1 | NR2C2AP | YWHAB | STX3 |
|  | TMEM9B | YWHAB | BTBD3 | LBR |
|  | XPO4 | LBR | SSR1 | FMR1 |
|  | BTBD3 | LRRC41 | PNPLA6 | C1orf144 |
|  | PDIA3 |  |  |  |
| **hsa-mir-15b** | TBL1XR1 | FUBP1 | WAPAL | CBARA1 |
|  | PHF20L1 | SIAH1 | CADM1 | PSME3 |
|  | SIAH1 | BAG5 | COPS7A | BAG5 |
|  | RANBP3 | DDX3X | WIPI2 | NUP50 |
|  | ATXN2 | USP14 | WSB1 | PPP2R1A |
|  | RAD23B |  |  |  |
| **hsa-miR-16** | KIF23 | PHF20L1 | SIAH1 | WIPI2 |
|  | C1orf9 | PCGF5 | BAG5 | PSME3 |
|  | PRKAR2A | RANBP3 | WIPI2 | CBARA1 |
|  | CDC14B | SRPR | USP14 | USP14 |
|  | SLC12A2 | SIAH1 | COPS7A | NUP50 |
|  | RAD23B | ATXN2 | WIPI2 | SPTLC1 |
|  | WAPAL | BAG5 | WSB1 | CSDE1 |
|  | TBL1XR1 |  |  |  |
| **hsa-mir-17** | APP | FASTK | NPLOC4 | SERP1 |
|  | ARID4B | HIF1A | OCRL | SLC16A9 |
|  | C1orf9 | MCL1 | RAB5B | SUV420H1 |
|  | CCNG2 | KIF23 | RNF145 | TMEM168 |
|  | CRK | TXNIP | RNF6 | TWF1 |
|  | USP46 | WAC | ZNF148 |  |
| **hsa-miR-181b** | ARL5A | TM9SF3 | DDX3X | GAPVD1 |
|  | BTBD3 | WSB1 | EIF4A2 | GLS |
|  | CAMK2G | ZNF207 | G3BP2 | LBR |
|  | CDC42BPA | MAT2A | PHF20L1 | TCERG1 |
|  | PICALM | NSMAF | RNF145 | XPO4 |
|  | PPP1CB | OSBPL8 |  |  |
| **hsa-mir-193b** | YWHAZ | TBL1XR1 | YWHAZ | LAMP2 |
|  | TMEM30A | MCL1 | CAPRIN1 |  |
| **hsa-miR-19b** | ACTN1 | MAT2A | CCNL1 | RAB5B |
|  | AKAP1 | OCRL | CDC42BPA | RNF145 |
|  | ARID4B | PITX1 | CLTC | RNF167 |
|  | ASNA1 | PRC1 | DDX3X | SLC6A8 |
|  | EXOC5 | FMR1 | ELAVL1 | SNX17 |
|  | FASTK | G3BP2 | GULP1 | TNPO2 |
|  | HNRNPF | IMPDH1 | WDR45L |  |
| **hsa-mir-20a** | APP | NPLOC4 | HIF1A | SUV420H1 |
|  | ARID4B | OCRL | KIF23 | TMEM168 |
|  | CCNG2 | RAB5B | MCL1 | TWF1 |
|  | CRK | SERP1 | TXNIP | USP46 |
|  | ZNF148 |  |  |  |
| **hsa-mir-20b** | APP | HIF1A | SERP1 | TXNIP |
|  | ARID4B | KIF23 | SLC16A9 | USP46 |
|  | C1orf9 | MCL1 | SUV420H1 | WAC |
|  | CCNG2 | NPLOC4 | TMEM168 | ZNF148 |
|  | CRK | OCRL | TWF1 | RAB5B |
|  | DNAJB6 | EIF4G2 | RNF145 | FASTK |
|  | RNF6 |  |  |  |
| **hsa-mir-25** | BSDC1 | G3BP2 | SUV420H1 | SRPR |
|  | DDX3X | NECAP1 | PPP1R12C | FMR1 |
|  | EXOC5 | NSMAF | G3BP2 |  |
| **hsa-miR-26a** | G3BP2 | LARP1 | SLC12A2 | NUS1 |
|  | C20orf24 | MCL1 | TMEM168 | RNF6 |
|  | CLASP2 | CLTC | EIF5 | SERP1 |
| **hsa-mir-34a** | ARID4B | C1orf9 | TBL1XR1 | ZNF207 |
| **hsa-mir-361-5p** | ZNF148 | TYW3 | SP1 | EXOC5 |
|  | RAD23B | DDX3X |  |  |
| **hsa-mir-425** | TOMM22 | FAR1 | PAPSS1 | SLC16A1 |
|  | CAB39 | PDLIM5 | EXOC5 | IQGAP1 |
|  | MANEA |  |  |  |
| **hsa-mir-494** | ARID4B | IGF2BP1 | PRPF4B | ZNF207 |
|  | EIF5 |  |  |  |
| **hsa-mir-503** | AP3D1 |  |  |  |
| **hsa-mir-532-5p** | SLC25A32 | TPM3 | DHX9 | ZKSCAN1 |
|  | PLEKHB2 | CDC14B | ZMPSTE24 |  |
| **hsa-mir-572** | CDC42SE2 | BRI3BP |  |  |
| **hsa-mir-574** | CLTC |  |  |  |
| **hsa-mir-575** | SLC25A24 | SURF4 | STOM |  |
| **hsa-mir-663** | ILK |  |  |  |
| **hsa-mir-665** | APLP2 | MANBAL | SMEK2 | TMED9 |
|  | DDX3X | MCRS1 | STX3 | TMEM129 |
|  | HMGB1 | SLC35B2 | TBL1XR1 | WDR48 |
| **hsa-mir-744** | UBE2L3 | TGFB1 |  |  |
| **hsa-mir-877** | CAP1 | CLTC | ARHGAP19 |  |
| **hsa-mir-885-3p** | SAFB | CHD7 | PFN1 | CFL1 |
| **hsa-mir-939** | TSC22D3 | C1orf144 | CNOT3 | WTAP |
|  | KDELR1 | BSDC1 |  |  |
| **hsa-mir-1228-5p** | HRAS | MPDU1 | ENSA | PICALM |
|  | TBX1 | APH1A | IGF2BP1 | SLC12A2 |
| **hsa-mir-149-3p** | LYPLA1 | EIF4G2 | TBL1XR1 | UBE2A |
|  | APP | LUC7L2 | TFDP1 | ZNF148 |
|  | BMI1 | MEF2C | TM9SF3 | ZNF207 |
|  | CHD7 | SEC23IP |  |  |
| **hsa-mir-150-3p** | PICALM | PDLIM5 |  |  |
| **hsa-mir-1908** | CRK | NKIRAS2 | PICALM |  |
| **hsa-mir-1909** | PJA2 | CAMK2G | LARP1 | TBL1XR1 |
|  | B3GNT2 | GRINA | NDRG3 | UBE2L3 |
|  | CADM1 | IGF2BP1 | PIGT | ZNF148 |
| **hsa-mir-1910** | MEF2C | TM9SF3 | AHCYL1 | FMR1 |
|  | KLHL13 |  |  |  |
| **hsa-mir-1915** | ATP6V0C | MAT2A | CHD7 | SPOP |
| **hsa-mir-1979** | HMGB1 | PFN1 | IGF2BP1 | SSR1 |
|  | ATP6V0C | TMEM129 | RNGTT |  |
| **hsa-mir-2861** | ACTN1 | TCEAL8 | MAT2A | SOX2 |
|  | ARL5A | EIF4A2 | RTN4 | XPO4 |
|  | CAB39 | GDI1 | SLC12A2 |  |
| **hsa-mir-3141** | ARFIP2 | C1orf107 | GNB2 | ACTN4 |
|  | BTBD3 | C1orf144 | HDAC8 | PATZ1 |
|  | APH1A | C20orf27 | MAZ | TNPO2 |
|  | BAT2 | CNOT3 | SLC6A8 |  |
| **hsa-mir-3172** | AP2M1 | SIAH1 | TBL1XR1 | TMEM30A |
|  | MCL1 | LAMP2 | YWHAZ | SP1 |
| **hsa-mir-3176** | LARP1 | GDI1 | MTA2 | C20orf27 |
|  | C1orf144 | HSPBP1 | NUS1 |  |
| **hsa-miR-3180-3p** | BSDC1 | SP1 | CAP1 | MRPL12 |
|  | FHL1 | BLMH | HMGB1 | SOX2 |
|  | G3BP2 | CAMK2G | IGF2BP1 | YWHAE |
| **hsa-mir-3185** | ABCD3 | HMGB1 | SERP1 | MAPK6 |
|  | DDX3X | IGF2BP1 | ZNF148 | RAB5C |
|  | TMED9 | WTAP |  |  |
| **hsa-mir-3187** | C1orf144 | PITX1 | GNB2 | NCSTN |
| **hsa-mir-3188** | ABCD3 | IGF2BP1 | MAPK6 | TMED9 |
|  | DDX3X | SERP1 | RAB5C | WTAP |
|  | HMGB1 | ZNF148 |  |  |
| **hsa-mir-3196** | ABCD3 | IGF2BP1 | MAPK6 | TMED9 |
|  | DDX3X | SERP1 | RAB5C | WTAP |
|  | HMGB1 | ZNF148 |  |  |
| **hsa-mir-3197** | MEF2C | CTNNB1 | BMI1 | KLHL9 |
|  | DHX9 | GLS | CLTC | MAT2A |
|  | TSPAN3 | LAMP2 | CSNK1E | RAB7A |
|  | ZNF503 | NAP1L3 | EIF4G2 | SERP1 |
|  | CAMK2G | PPP1CB | FMR1 | ZNF24 |
|  | CAPZA1 | WDR48 |  |  |
| **hsa-mir-4270** | DDX3X | ARID4B | PSME3 | TOMM22 |
|  | TM9SF3 | FMR1 | RANBP3 | WTAP |
|  | ZNF148 | PJA2 | TMEM30A |  |
| **hsa-mir-4281** | IGF2BP1 | ALCAM | PAPSS1 | C1orf9 |
|  | SLC6A8 | APP | RAB5B | HDAC8 |
|  | IPO11 | BAG5 | SERPINH1 | PCGF5 |
|  | RAB7A | FMR1 | SMEK2 | SEPHS2 |
|  | SLC16A1 | G3BP2 | WAPAL | SMARCE1 |
|  | WDR48 | GNB2 | ZNF148 | ZNF207 |
|  | ARID4B | H1F0 | ZYX |  |
| **hsa-mir-4322** | BTBD3 | HDAC8 | ZYX | IGF2BP1 |
|  | EIF4G1 | PRPF4B | DLST |  |
| **hsa-mir-532-5p** | ARPC4 |  |  |  |
| **hsa-mir-762** | ABCD3 | IGF2BP1 | MAPK6 | TMED9 |
|  | DDX3X | SERP1 | RAB5C | WTAP |
|  | HMGB1 | ZNF148 |  |  |
| **hsa-mir-92b-5p** | C1orf128 | MEF2C | DDX3X | PJA2 |
|  | TNPO3 | UBE2L3 | HEXA | TCERG1 |
|  | ZFR | YWHAZ | IMPDH1 | WTAP |
|  | PRPF4B | ARID4B | PARVA | ZNF275 |

**24 hours post infection**

| **Upregulated miRNAs** | **Downregulated Genes** | | | |
| --- | --- | --- | --- | --- |
| **hsa-let-7a** | ACVR1B | CCNJ | COL14A1 | DZIP1 |
|  | APPBP2 | CDC25A | COL5A2 | EDEM3 |
|  | C10orf140 | CHD7 | CPEB2 | EIF2C3 |
|  | CALD1 | CHD9 | CTDSPL2 | FAM135A |
|  | PLCB4 | NAPEPLD | LCORL | FAM84B |
|  | SLC25A27 | NCOA3 | LRRFIP1 | GDPD1 |
|  | SNX16 | NID2 | LSM11 | GNPTAB |
|  | SP8 | NIPA1 | MEF2D | IGF2BP2 |
|  | SYT11 | ONECUT2 | NAB1 | KLF9 |
|  | TMEM65 | USP32 | VASH2 |  |
| **hsa-let-7b** | ACVR1B | COL14A1 | GNPTAB | NID2 |
|  | APPBP2 | COL5A2 | IGF2BP2 | NIPA1 |
|  | C10orf140 | CPEB2 | KLF9 | ONECUT2 |
|  | C6orf211 | CTDSPL2 | LCORL | PLCB4 |
|  | CALD1 | DZIP1 | LSM11 | SLC25A27 |
|  | CCNJ | EDEM3 | MEF2D | SNX16 |
|  | CDC25A | FAM135A | NAB1 | SP8 |
|  | CHD7 | FAM84B | NAPEPLD | SYT11 |
|  | CHD9 | GDPD1 | NCOA3 | TMEM65 |
|  | USP32 | VASH2 |  |  |
| **hsa-let-7c** | ACVR1B | CDC25A | CPEB2 | FAM135A |
|  | APPBP2 | CHD7 | CTDSPL2 | FAM84B |
|  | C10orf140 | CHD9 | DZIP1 | GDPD1 |
|  | CALD1 | COL14A1 | EDEM3 | GNPTAB |
|  | CCNJ | COL5A2 | EIF2C3 | IGF2BP2 |
|  | KLF9 | MEF2D | NCOA3 | ONECUT2 |
|  | LCORL | NAB1 | NID2 | PLCB4 |
|  | LSM11 | NAPEPLD | NIPA1 | SLC25A27 |
|  | SNX16 | SYT11 | USP32 | VASH2 |
|  | SP8 | TMEM65 |  |  |
| **hsa-let-7e** | ACVR1B | CDC25A | CPEB2 | FAM135A |
|  | APPBP2 | COL5A2 | CTDSPL2 | FAM84B |
|  | C10orf140 | CHD7 | DZIP1 | GNPTAB |
|  | CALD1 | CHD9 | EDEM3 | IGF2BP2 |
|  | CCNJ | COL14A1 | EIF2C3 | KLF9 |
|  | LCORL | MEF2D | NCOA3 | ONECUT2 |
|  | LRRFIP1 | NAB1 | NID2 | PLCB4 |
|  | LSM11 | NAPEPLD | NIPA1 | SLC25A27 |
|  | SP8 | SYT11 | VASH2 | SNX16 |
|  | USP32 |  |  |  |
| **hsa-miR-103** | BLMH | CDK6 | KIAA1033 | PHF20L1 |
|  | BSDC1 | DICER1 | KIF23 | PI4KB |
|  | CAB39 | EIF5 | PCGF5 | SMARCE1 |
| **hsa-mir-106a** | CRK | NPLOC4 | SERP1 | WAC |
|  | MCL1 | OCRL | SUV420H1 | ZNF148 |
| **hsa-mir-106b** | ANKRD13C | EIF4G2 | MCL1 | SERP1 |
|  | ARID4B | FASTK | NPLOC4 | SUV420H1 |
|  | CRK | HIF1A | RAB5B | TMEM168 |
|  | USP46 | KIF23 | RNF6 | TWF1 |
|  | WAC | ZNF148 |  |  |
| **hsa-mir-107** | BLMH | DICER1 | KIF23 | PI4KB |
|  | BSDC1 | KIAA1033 | PCGF5 | SMARCE1 |
|  | CAB39 | EIF5 | PHF20L1 | TWF1 |
|  | CDK6 |  |  |  |
| **hsa-mir-10a** | USP46 | TMEM183A | CAMK2G | CADM1 |
| **hsa-mir-1202** | ARID4B | TCEAL8 | DSP | MLEC |
|  | SLMAP | RALY | CNOT3 | CLASP2 |
|  | MTA2 | BRMS1 |  |  |
| **hsa-mir-1207-5p** | CBX5 | RBM14 | GNB2 | MCL1 |
|  | SH3GLB2 | MCRS1 | IMPDH1 | CDK2AP2 |
|  | DUSP3 | C20orf29 | TAF9B | CCDC86 |
|  | PBX1 | CNOT3 | EPHX1 | AK1 |
|  | PRPF4 | USP46 | LOXL1 | C20orf27 |
|  | OSBPL8 |  |  |  |
| **hsa-mir-1224-5p** | UBAP2L | DDX3X | TAF9B | MLEC |
|  | ZNF275 | ATRX | CELF2 | SP1 |
|  | CDK6 | TSTA3 |  |  |
| **hsa-mir-1225-5p** | CDC14B | SNRPA | TSPAN3 | CLSPN |
|  | PABPC4 | CSK |  |  |
| **hsa-mir-1228-5p** | HRAS | MPDU1 | ENSA | PICALM |
|  | TBX1 | APH1A | IGF2BP1 | SLC12A2 |
| **hsa-mir-1231** | SNRPA | SEC23IP | CLTC | ZNF148 |
|  | ARID4B |  |  |  |
| **hsa-mir-125a-3p** | BRCA1 | GANAB | PPP1CB | FLNA |
|  | TYW3 | MTMR14 |  |  |
| **hsa-miR-125b-1-3p** | NKX2-5 |  |  |  |
| **hsa-mir-1271** | DLAT | WIPI2 | NUP50 | IGF2BP1 |
|  | JMJD1C | EIF5 | RAD23B | BSDC1 |
|  | PROSC | STARD7 | PPP1R12C | SNX17 |
|  | GJC1 | ELAVL1 | CDC42BPA | PFN1 |
|  | PHF20L1 | UBE2L3 | DEXI | SLC35C1 |
|  | PCGF5 | TBX1 | CAPNS1 | RDX |
|  | NUS1 | FMR1 | AHCYL1 | PRC1 |
|  | CFL1 | KDELR1 |  |  |
| **hsa-mir-1308** | OSBPL8 | CHD7 | UBE2A | PCBP2 |
| **hsa-miR-135a-3p** | PARVA |  |  |  |
| **hsa-mir-140-3p** | UBAP2L | YWHAZ | PCGF5 | CLSPN |
|  | RBFOX2 | ARF5 | CCNL2 | CELF2 |
|  | SUV420H1 | HYOU1 | TSPAN3 | PPP1CB |
|  | HS6ST2 | CAPRIN1 | NKIRAS2 | PI4KB |
|  | ATXN7L3B | HOXB5 | ZNF24 | TADA3 |
|  | APLP2 | LARP1 | CDK6 |  |
| **hsa-mir-149** | YWHAZ | SP1 | PRKAR2A | CCNI |
|  | USP10 | YWHAZ | BTBD3 | EXOC5 |
|  | TEX261 | TOP1 |  |  |
| **hsa-mir-149-3p** | LYPLA1 | LUC7L2 | UBE2A | BMI1 |
|  | SLMAP | EIF4G2 | JMJD1C | CHD7 |
|  | TM9SF3 | MEF2C | TFDP1 | SEC23IP |
|  | ZNF148 | ZNF207 |  |  |
| **hsa-mir-150-3p** | PICALM | PDLIM5 |  |  |
| **hsa-mir-151-5p** | WIPI2 |  |  |  |
| **hsa-mir-152** | AKAP1 | CHD7 | DICER1 | FMR1 |
|  | ARL6IP1 | NR2C2AP | BTBD3 | C1orf144 |
|  | TMEM9B | YWHAB | SSR1 | LRRC41 |
|  | XPO4 | LBR | PPP1CB | PNPLA6 |
|  | BTBD3 | YWHAB | LBR | PDIA3 |
| **hsa-mir-15b** | PRKAR2A | RANBP3 | DDX3X | WIPI2 |
|  | C1orf9 | ATXN2 | USP14 | WSB1 |
|  | KIF23 | RAD23B | WAPAL | CBARA1 |
|  | SLC12A2 | FUBP1 | CADM1 | PSME3 |
|  | PHF20L1 | BAG5 | COPS7A | PPP2R1A |
|  | NUP50 |  |  |  |
| **hsa-miR-16** | KIF23 | C1orf9 | NUP50 | RAD23B |
|  | ATXN2 | CBARA1 | PCGF5 | RANBP3 |
|  | BAG5 | CDC14B | PHF20L1 | SLC12A2 |
|  | WIPI2 | COPS7A | PRKAR2A | SRPR |
|  | WSB1 | CSDE1 | PSME3 | USP14 |
| **hsa-mir-17** | ANKRD13C | C1orf9 | FASTK | KIF23 |
|  | ARID4B | CRK | HIF1A | MCL1 |
|  | NPLOC4 | RNF145 | SUV420H1 | USP46 |
|  | OCRL | RNF6 | TMEM168 | WAC |
|  | RAB5B | SERP1 | TWF1 | ZNF148 |
| **hsa-miR-181b** | ANKRD13C | ARL5A | CAMK2G | DDX3X |
|  | ARL5A | BTBD3 | CDC42BPA | EIF4A2 |
|  | GAPVD1 | MAP1B | PHF20L1 | G3BP2 |
|  | GLS | MAT2A | PICALM | TM9SF3 |
|  | HSP90B1 | NSMAF | PPP1CB | WSB1 |
|  | LBR | OSBPL8 | RNF145 | ZNF207 |
|  | TCERG1 |  |  |  |
| **hsa-mir-182** | ARHGDIA | NCKAP1 | PHF20L1 | FMR1 |
|  | CDC27 | ELAVL1 | RASA1 | KDELR1 |
|  | CFL1 | OCRL | ZMPSTE24 | MAP1LC3B |
|  | EIF5 | PCGF5 | RDX | MEF2C |
|  | RTN4 | SLC35C1 | SOX2 | WIPI2 |
| **hsa-mir-18a** | C1orf9 | HIF1A | PHF20L1 | ALCAM |
| **hsa-mir-18a-3p** | PSME3 | STIP1 | FMR1 | RAB7A |
|  | RBM14 |  |  |  |
| **hsa-mir-1908** | CRK | NKIRAS2 | PICALM |  |
| **hsa-mir-1909** | PJA2 | GRINA | NDRG3 | UBE2L3 |
|  | CADM1 | IGF2BP1 | PIGT | ZNF148 |
|  | CAMK2G | LARP1 | SPAG9 |  |
| **hsa-mir-1909-5p** | BAZ1A | MEF2C | ZNF24 |  |
| **hsa-mir-1910** | MEF2C | AHCYL1 | KLHL13 | SRRM2 |
|  | TM9SF3 | FMR1 |  |  |
| **hsa-mir-1915** | ATP6V0C | MAT2A | CHD7 | SPOP |
| **hsa-mir-193b** | YWHAZ | MCL1 | YWHAZ | CAPRIN1 |
|  | LAMP2 |  |  |  |
| **hsa-miR-196a** | SSR1 |  |  |  |
| **hsa-mir-19a** | ACTN1 | CDC42BPA | G3BP2 | PRC1 |
|  | AKAP1 | CLTC | GULP1 | RNF145 |
|  | ARID4B | DDX3X | HNRNPF | SLC6A8 |
|  | ASNA1 | DICER1 | IMPDH1 | SNX17 |
|  | ATRX | ELAVL1 | MAPK6 | TNPO2 |
|  | CAB39 | EXOC5 | OCRL | WDR45L |
|  | CCNL1 | FMR1 | PCGF5 |  |
| **hsa-mir-20a** | ANKRD13C | NPLOC4 | TWF1 | SUV420H1 |
|  | ARID4B | OCRL | USP46 | TMEM168 |
|  | CRK | RAB5B | ZNF148 | MCL1 |
|  | HIF1A | SERP1 | KIF23 |  |
| **hsa-mir-20b** | ANKRD13C | EIF4G2 | NPLOC4 | RNF6 |
|  | ARID4B | FASTK | OCRL | SERP1 |
|  | C1orf9 | HIF1A | RAB5B | SUV420H1 |
|  | CRK | KIF23 | RNF145 | TMEM168 |
|  | DNAJB6 | MCL1 | USP46 | TWF1 |
|  | WAC | ZNF148 |  |  |
| **hsa-mir-222** | ARID1A | FMR1 | MAT2A |  |
| **hsa-mir-2277** | MEF2C | AHCYL1 | KLHL13 | SRRM2 |
|  | TM9SF3 | FMR1 |  |  |
| **hsa-mir-23a** | C1orf9 | HSP90B1 | PHF20L1 | RAD23B |
|  | CAB39 | LBR | PPIF | SEC23A |
|  | CHD7 | MAT2A | PPP1CB | SMEK2 |
|  | G3BP2 | MEF2C | PPP1CB | TMPO |
|  | G3BP2 | NUP50 | PROSC | TOP1 |
|  | GLS | NUS1 | PRPF4B | WSB1 |
|  | HS6ST2 | OSBPL8 |  |  |
| **hsa-mir-23b** | G3BP2 | HS6ST2 | OSBPL8 | RAD23B |
|  | C1orf9 | LBR | PROSC | SEC23A |
|  | CAB39 | MAT2A | PHF20L1 | SMEK2 |
|  | FMR1 | MEF2C | PPIF | TMPO |
|  | G3BP2 | NUP50 | PPP1CB | TOP1 |
|  | GLS | NUS1 | PRPF4B | USP46 |
| **hsa-miR-24** | SP1 | PHF20L1 | RASA1 | ZXDC |
|  | TOP1 | PCGF5 | RAB5B | EXOC5 |
|  | XPO4 | GPX3 | DHFR |  |
| **hsa-mir-25** | ATRX | EXOC5 | NECAP1 | SPRYD4 |
|  | BSDC1 | FMR1 | NSMAF | SRPR |
|  | DDX3X | G3BP2 | PPP1R12C | SUV420H1 |
| **hsa-miR-26a** | C20orf24 | EIF5 | MCL1 | SLC12A2 |
|  | CDK6 | LARP1 | NUS1 | TMEM168 |
|  | CLASP2 | G3BP2 | RNF6 | TMEM68 |
|  | CLTC | MAP1B | SERP1 |  |
| **hsa-mir-2861** | ACTN1 | EIF4A2 | RTN4 | TCEAL8 |
|  | ARL5A | GDI1 | SLC12A2 | TOP1 |
|  | CAB39 | MAT2A | SOX2 | XPO4 |
| **hsa-mir-297** | TCERG1 | SP1 | DDX3X |  |
| **hsa-mir-3148** | ALCAM | GNB2 | PCGF5 | SMARCE1 |
|  | ARID4B | H1F0 | RAB5B | SMEK2 |
|  | BAG5 | HDAC8 | RAB7A | WAPAL |
|  | C1orf9 | IGF2BP1 | SEPHS2 | WDR48 |
|  | FAHD1 | IPO11 | SERPINH1 | ZNF148 |
|  | FMR1 | JMJD1C | SLC16A1 | ZNF207 |
|  | G3BP2 | PAPSS1 | SLC6A8 | ZYX |
| **hsa-mir-3176** | LARP1 | GDI1 | MTA2 | C20orf27 |
|  | C1orf144 | HSPBP1 | NUS1 |  |
| **hsa-miR-3180-3p** | LARP1 | GDI1 | MTA2 | C20orf27 |
|  | C1orf144 | HSPBP1 | NUS1 |  |
| **hsa-mir-3185** | ABCD3 | IGF2BP1 | MAPK6 | TOP1 |
|  | DDX3X | SERP1 | RAB5C | WTAP |
|  | HMGB1 | ZNF148 | TMED9 |  |
| **hsa-mir-3187** | C1orf144 | PITX1 | GNB2 | NCSTN |
| **hsa-mir-3188** | ABCD3 | IGF2BP1 | MAPK6 | TOP1 |
|  | DDX3X | SERP1 | RAB5C | WTAP |
|  | HMGB1 | ZNF148 | TMED9 |  |
| **hsa-mir-3196** | ABCD3 | IGF2BP1 | MAPK6 | TOP1 |
|  | DDX3X | SERP1 | RAB5C | WTAP |
|  | HMGB1 | ZNF148 | TMED9 |  |
| **hsa-mir-3197** | ATRX | LAMP2 | DHX9 | SLMAP |
|  | BMI1 | MAT2A | EIF4G2 | TOP1 |
|  | CAMK2G | MEF2C | FMR1 | TSPAN3 |
|  | CAPZA1 | NAP1L3 | GLS | WDR33 |
|  | CLTC | PPP1CB | KLHL9 | WDR48 |
|  | CSNK1E | RAB7A | ZNF503 | ZNF24 |
|  | CTNNB1 | SERP1 |  |  |
| **hsa-mir-339-3p** | YWHAE |  |  |  |
| **hsa-mir-342-3p** | WDR77 | RASA1 | SLC6A8 |  |
| **hsa-mir-345** | USP46 |  |  |  |
| **hsa-mir-346** | TMEM9B | C1orf144 |  |  |
| **hsa-mir-361-5p** | ZNF148 | TYW3 | EXOC5 | SP1 |
|  | RAD23B | DDX3X |  |  |
| **hsa-mir-362-5p** | RNF145 |  |  |  |
| **hsa-mir-425** | TOMM22 | FAR1 | PAPSS1 | SLC16A1 |
|  | CAB39 | PDLIM5 | EXOC5 | IQGAP1 |
|  | MANEA |  |  |  |
| **hsa-mir-4270** | DDX3X | TM9SF3 | FMR1 | RANBP3 |
|  | ATRX | ZNF148 | PJA2 | TOMM22 |
|  | DICER1 | ARID4B | PSME3 | WTAP |
| **hsa-mir-4281** | ALCAM | SMARCE1 | GNB2 | PCGF5 |
|  | ARID4B | SMEK2 | H1F0 | RAB5B |
|  | BAG5 | WAPAL | HDAC8 | RAB7A |
|  | C1orf9 | WDR48 | IGF2BP1 | SEPHS2 |
|  | FAHD1 | ZNF148 | IPO11 | SERPINH1 |
|  | FMR1 | ZNF207 | JMJD1C | SLC16A1 |
|  | G3BP2 | ZYX | PAPSS1 | SLC6A8 |
| **hsa-mir-4284** | XPO4 | GRINA | MTMR14 | RAB5B |
|  | C20orf27 | RAB5C | CSK |  |
| **hsa-mir-4299** | SUV420H1 | ZNF148 | HIF1A | PRPF4B |
|  | LYPLA1 | SLC6A8 | PHF20L1 | MAPK6 |
|  | USP10 | DEK | YIPF5 | CADM1 |
|  | JMJD1C | CLASP2 |  |  |
| **hsa-mir-4322** | ANKRD13C | EIF4G1 | PRPF4B | DLST |
|  | BTBD3 | HDAC8 | ZYX | IGF2BP1 |
| **hsa-mir-494** | ARID4B | IGF2BP1 | PRPF4B | ZNF207 |
|  | EIF5 |  |  |  |
| **hsa-mir-503** | AP3D1 | PLEKHB2 | CDK6 | ZMPSTE24 |
|  | SLC25A32 | C9orf123 | CDC14B | DEK |
|  | KIAA1033 | TPM3 | DHX9 | ZKSCAN1 |
| **hsa-mir-532-5p** | SLMAP | ARPC4 |  |  |
| **hsa-mir-572** | BRI3BP |  |  |  |
| **hsa-mir-574-5p** | CHD7 | ELAVL1 | NSD1 | PRPF4B |
|  | TNPO2 |  |  |  |
| **hsa-mir-575** | SLC25A24 | STOM |  |  |
| **hsa-mir-602** | WAC |  |  |  |
| **hsa-mir-663** | ILK |  |  |  |
| **hsa-mir-663b** | CCNL2 | ARHGDIA | SLC6A8 |  |
| **hsa-mir-665** | WDR48 | TMED9 | MCRS1 | MANBAL |
|  | HOXB5 | APLP2 | SMEK2 | HMGB1 |
|  | ANKRD13C | SLC35B2 | DDX3X |  |
| **hsa-mir-675** | DICER1 | LUC7L2 | MAT2A | RALGDS |
| **hsa-mir-744** | UBE2L3 | TGFB1 |  |  |
| **hsa-mir-760** | FMR1 |  |  |  |
| **hsa-mir-762** | ABCD3 | IGF2BP1 | MAPK6 | TOP1 |
|  | DDX3X | SERP1 | RAB5C | WTAP |
|  | HMGB1 | ZNF148 | TMED9 |  |
| **hsa-mir-766** | IMP3 | C16orf63 | C1orf212 | HNRNPF |
|  | LUC7L2 | ZNF559 | MFN2 | UBE4A |
|  | MED18 | SLC35A4 | IGF2BP1 | DDX17 |
| **hsa-mir-877** | CAP1 | CLTC | ARHGAP19 |  |
| **hsa-mir-885-3p** | CDK6 | SAFB | CHD7 | PFN1 |
|  | CFL1 |  |  |  |
| **hsa-mir-92b-5p** | ARID4B | IMPDH1 | PRPF4B | WTAP |
|  | C1orf128 | MEF2C | TCERG1 | YWHAZ |
|  | DDX3X | PARVA | TNPO3 | ZFR |
|  | HEXA | PJA2 | UBE2L3 | ZNF275 |
| **hsa-mir-939** | KDELR1 | BSDC1 | CNOT3 | WTAP |
|  | C1orf144 |  |  |  |
